# Coverage of procedures related to chronic kidney disease care in the Brazilian Unified Health System (SUS): analysis of the 2015–2024 decade

**DOI:** 10.1590/2175-8239-JBN-2025-0144en

**Published:** 2026-01-23

**Authors:** Farid Samaan, Lan Hee Suh, Sônia Dias Lanza Freire, Ricardo Sesso, Gianna Mastroianni Kirsztajn

**Affiliations:** 1 Secretaria de Estado da Saúde de São Paulo, Coordenadoria de Regiões de Saúde, Grupo de Planejamento e Avaliação, São Paulo, SP, Brazil.; 2 Instituto Dante Pazzanese de Cardiologia, Divisão de Pesquisa, São Paulo, SP, Brazil.; 3 Universidade Federal de São Paulo, Disciplina de Nefrologia, São Paulo, SP, Brazil.

**Keywords:** Unified Health System, Access to Health Services, Health Information Systems, Kidney Replacement Therapy, Chronic Kidney Disease, Hemodialysis, Arteriovenous Fistula

## Abstract

**Introduction:**

Globally, quantitative information on healthcare coverage for chronic kidney disease (CKD) is scarce. Our objective was to estimate the supply/demand ratio for CKD-related procedures in the Brazilian Unified Health System (SUS) between 2015 and 2024.

**Methods:**

The volume of tests, consultations and treatments related to CKD was retrieved from the website of the SUS Information Technology Department. The requirement parameters of these procedures were obtained from the Ministry of Health ordinances as well as from literature review. The percentage coverage of each procedure was defined by the ratio between the volume performed and the estimated need.

**Results:**

Coverage of the following procedures increased between 2015 and 2024: serum creatinine dosage (70% to 122%), proteinuria testing (4% to 12%), kidney ultrasonography (76% to 107%), outpatient consultation with a nephrologist (48% to 164%), multidisciplinary care of pre-dialysis CKD (0% to 3%), and chronic dialysis (69% to 81%). Coverage of kidney biopsy remained nearly stable (19% to 21%). There was a reduction in coverage of arteriovenous fistula for hemodialysis (HD) (66% to 59%) and of kidney transplantation (46% to 37%). The use of peritoneal dialysis (PD) among chronic dialysis methods (PD and HD) declined from 7% to 4%.

**Conclusion:**

Possible explanations for these results include excessive creatinine testing and nephrology consultations, neglect of CKD screening for proteinuria, lack of adherence to multidisciplinary pre-dialysis follow-up, underutilization of PD, and insufficient availability of kidney biopsy and kidney replacement therapy (lower coverage of kidney transplantation compared to chronic dialysis).

## Introduction

Chronic kidney disease (CKD) is one of the best models for health prevention, since its main risk factors are well-known (hypertension, diabetes mellitus, aging, obesity), early diagnosis is simple and low-cost, and many primary, secondary, and tertiary prevention measures are feasible and cost-effective^1,2^.

Paradoxically, CKD is the most neglected chronic noncommunicable disease worldwide, especially in low- and middle-income countries^3^. Among these nations, Brazil stands out — a Latin American country undergoing rapid epidemiological and demographic transition, with the fourth largest population in the world undergoing chronic dialysis^4^. In recent decades, the prevalence of major CKD risk factors has increased significantly in Brazil: obesity, from 12% to 23%; hypertension (HTN), from 20% to 28%; diabetes (DM), from 5% to 8%; and age over 60, from 9% to 13%^5,6^.

The global agenda for tackling CKD involves not only professional training and patient awareness campaigns, but also expanding healthcare coverage^1,3^. For this latter action, determining the estimated needs of each population and monitoring the provision of healthcare services is essential.

In Brazil, the methodology for calculating service needs within the Unified Health System (*Sistema Único de Saúde* or SUS) was based on national and international scientific evidence, expert opinions, and public consultation, culminating in the Ordinance on Care Parameters for Planning and Programming Health Actions and Services in the SUS^7^. Globally, quantitative information on CKD care coverage is scarce. Thus, the aim of this study was to estimate the supply/demand ratio for CKD-related procedures in the Brazilian Unified Health System (SUS) between 2015 and 2024.

## Methods

### Study Design

This is a descriptive study based on the outpatient (SIA/SUS) and hospital (SIH/SUS) information systems of the SUS^8^. The SIA and SIH are secondary databases that store information on healthcare-related procedures (consultations, tests, high-cost medications, surgeries and other therapies, hospital admissions, among others). These systems have a national coverage, encompassing all healthcare facilities accredited to provide services to the SUS, and are designed for billing purposes. Information on the number of procedures performed is submitted monthly, electronically, by healthcare facilities to the Ministry of Health, which consolidates and publishes the data on the DATASUS website within 60–90 days. These data are publicly available and allow for stratification by municipality, micro and macro health regions of the states and regions of Brazil.

The analysis covered the period from January 1, 2015, to December 31, 2024, and was conducted in Brazil, according to geographic regions. Data extraction from information systems was performed between January and March 2025. This study is exempt from review by a Research Ethics Committee, as it uses exclusively secondary, publicly available data, with grouped information and no possibility of accessing individual-level data.

### Variables of Interest

The procedures related to CKD care referred to in this study were: serum creatinine measurement, urine albumin measurement, 24-hour proteinuria assessment, kidney ultrasound, nephrology consultation, kidney biopsy, multidisciplinary CKD care, arteriovenous fistula creation, hemodialysis, peritoneal dialysis, and kidney transplantation^8^. These procedures were selected according to the following criteria: scientific relevance, availability of information in secondary SUS databases, and the presence of estimated needs in current guidelines, ministerial ordinances, or literature reviews (Table 1)^7,8,9,10,11,12^. A detailed list of these procedures, along with their respective codes in the SUS Management System for the Table of Procedures, Medications, and OPM (Orthotics, Prosthetics, and Special Materials) (SIGTAP, in Portuguese)^13^, is provided in Table S1.

**Table 1:** Parameters for procedure requirements related to chronic kidney disease care

Procedure		Requirements parameter
Ancillary tests	Serum creatinine measurement	1 test/year per adult with hypertension and/or diabetes mellitus^9^
	Proteinuria measurement	Sum of albuminuria and proteinuria measurements = 1 test/year per adult with hypertension and/or diabetes^9^
	Kidney ultrasound	800 tests/year per 100,000 inhabitants^7^
	Kidney biopsy	10 biopsies/year per 100,000 inhabitants^10^
Consultations	Nephrology consultation	1,600 consultations/year per 100,000 inhabitants^7^
	Multidisciplinary CKD care	5 procedures/year per adult with stage 4 or 5 non-dialytic CKD^7,13^
Surgical procedures	Arteriovenous fistula for hemodialysis	Number of AVF created/month = 5% of the number of patients on hemodialysis^11^
Kidney replacement therapy	Chronic dialysis (hemodialysis and peritoneal dialysis)	Prevalence of chronic dialysis patients among those aged ≥20 years, by region in Brazil: North: 0.08%; Northeast: 0.11%; Southeast: 0.13%; South: 0.11%; Midwest: 0.13%; Brazil: 0.12%^7^
	Kidney transplantation	Number of kidney transplants/year = 10% of the number of prevalent patients on chronic dialysis^12^

Abbreviations – CKD: chronic kidney disease; AVF: arteriovenous fistula.

The coverage percentage for each procedure was defined as the ratio between the actual amount and the estimated need. Coverage results for the exams, consultations, and treatments evaluated were calculated annually and stratified according to geographic region in Brazil. The variation (Δ) between the volume performed and the coverage of each procedure was calculated at two points in time (2015 and 2024), relatively (relative Δ, in %), and using the following formula:

Δ (%) = (2024 result – 2015 result)*100/2015 result

### Reference Population

The reference population for coverage estimates of serum creatinine dosage, proteinuria, kidney ultrasound, consultations with nephrologists, kidney biopsy, and multidisciplinary CKD care consisted of individuals who rely exclusively on the SUS. This population was calculated as the difference between the total population and the percentage of supplementary health coverage in each region of Brazil^14^.

The reference population used to estimate chronic dialysis and kidney transplant coverage consisted of the number of individuals who rely exclusively on the SUS, plus the percentage of the population with supplementary health insurance who accessed the SUS for these procedures in each region of Brazil. This correction factor was estimated based on reimbursement data from the National Regulatory Agency for Supplementary Health (ANS, in Portuguese)^14,15^ (Table S2). For chronic dialysis, the use of SUS by beneficiaries of private health insurance plans was estimated at: 60% in the North Region, 25% in the Northeast, 20% in the Southeast and South, 15% in the Midwest, and 20% in Brazil overall. For kidney transplantation, the estimated percentage was: 90% in the North Region, 70% in the Northeast, 40% in the Southeast and South, 45% in the Midwest, and 45% in Brazil as a whole.

The adult population with HTN and/or diabetes mellitus, used in the analysis of serum creatinine and proteinuria coverage, was estimated at 30%, based on prevalences of 29% for HTN, 9% for diabetes mellitus, and 8% for both conditions in combination^5^. Individuals with stage 4 and 5 non-dialysis CKD were estimated at 0.2% of the adult population^7^.

The reference population for estimating arteriovenous fistula coverage was the number of individuals undergoing hemodialysis. The mean number of prevalent dialysis patients (HD and PD) each year was estimated based on the number of procedures performed in the SUS^8^ (Table S3). Specifically, peritoneal dialysis (PD) utilization in the SUS (%) was estimated using the formula: number of individuals on PD*100/total number of individuals on chronic dialysis (HD + PD).

### Statistical Analysis

The variables of interest, extracted from the SUS information systems, were recorded in a spreadsheet program (Microsoft Excel software, version 2019). In the same program, the number of adults with HTN and/or DM, the number of procedures required (according to pre-established parameters), and the respective coverage (supply-to-need ratios) were calculated.

## Results

Between 2015 and 2024, the total Brazilian population increased from 202,403,642 to 212,583,750, corresponding to a 5.0% growth. The largest population increase was observed in the Midwest Region (+10.8%) and the smallest was in the Northeast Region (+3.5%). During the same period, the number of inhabitants aged 20 years or older increased by 11.3% in Brazil, with the highest increase in the North Region (+18.6%) and the lowest in the Southeast Region (+8.8%). The population aged ≥ 60 years increased by 37.1% in Brazil, with the highest increase in the Midwest Region (+48.6%) and the lowest in the Northeast Region (+32.7%). Supplementary health coverage in Brazil showed a slight decline between 2015 and 2024, decreasing from 24.7% to 24.0% (a reduction of 2.8%). The greatest reductions occurred in the North (−10.5%) and Southeast (−7.8%) regions (Table S4).

Between 2015 and 2024, the number of serum creatinine tests increased from 31,341,496 to 55,671,370 (+77.6%); the largest relative increase occurred in the North Region (+110.1%) and the smallest in the Southeast Region (+66.7%). During the same period, the number of proteinuria measurements (urinary albumin and 24-hour proteinuria) increased from 1,246,144 to 4,069,988 (+226.6%); the largest relative increase was in the Southeast Region (+316.3%) and the smallest in the North Region (+66.4%). The number of kidney ultrasounds increased from 928,175 to 1,384,854 (+49.2%); the largest increase occurred in the Northeast Region (+92.7%) and the smallest in the South Region (+30.8%). Between 2015 and 2024, nephrology consultations increased from 1,159,174 to 4,246,100 (a 266.3% increase); the highest relative increase was in the Northeast Region (+535.0%) and the lowest in the North Region (+72.2%). In the same period, the number of kidney biopsies increased from 2,834 to 3,393 (+19.7%); the largest increase occurred in the North Region (+218.2%), whereas the Midwest Region recorded a decline (−25.3%). There were 34,430 arteriovenous fistulas created in 2015 and 40,334 in 2024 (+17.1%); the greatest relative increase was in the North Region (+57.0%) and the smallest in the South Region (+1.6%). The number of kidney transplantations performed in 2015 was 4,925 and in 2024, 5,065 (+2.8%); the largest relative increase occurred in the Midwest Region (+112.7%), and two regions experienced a decline in this number: Northeast (−5.9%) and Southeast (−2.1%) (Table 2).

**Table 2:** Number of procedures related to CKD care funded by the SUS, by region in Brazil (2015–2024)

Procedure (n) Region	2015	2016	2017	2018	2019	2020	2021	2022	2023	2024	Rel. Δ (%)
Serum creatinine											
North	1,959,000	2,004,077	2,060,957	2,280,767	2,469,609	2,172,184	3,021,523	3,569,288	3,867,777	4,116,055	+110.1
Northeast	5,980,782	6,043,061	6,588,021	7,287,783	7,809,865	6,453,628	8,553,296	9,622,308	10,542,531	11,501,585	+92.3
Southeast	16,457,313	16,703,581	16,883,552	18,712,399	19,925,259	16,607,054	20,816,569	23,181,565	25,733,498	27,441,005	+66.7
South	4,915,068	5,077,338	5,421,119	5,815,820	6,268,103	5,093,574	6,164,617	7,125,974	7,978,346	8,486,227	+72.7
Midwest	2,029,333	2,180,719	2,422,533	2,454,803	2,852,839	2,310,253	3,040,985	3,566,131	3,837,401	4,126,498	+103.3
Total	31,341,496	32,008,776	33,376,182	36,551,572	39,325,675	32,636,693	41,596,990	47,065,266	51,959,553	55,671,370	+77.6
Proteinuria											
North	126,219	150,350	158,877	141,461	148,971	144,750	163,753	166,378	168,571	209,994	+66.4
Northeast	177,701	179,294	193,342	219,746	278,000	235,802	313,358	356,767	424,323	496,200	+179.2
Southeast	532,648	627,917	650,542	781,909	920,051	761,313	1,000,185	1,250,417	1,568,906	2,217,237	+316.3
South	289,488	285,489	306,658	362,521	422,037	367,553	471,259	606,577	779,328	916,493	+216.6
Midwest	120,088	105,055	136,788	149,037	154,748	127,414	181,539	189,128	218,344	230,064	+91.6
Total	1,246,144	1,348,105	1,446,207	1,654,674	1,923,807	1,636,832	2,130,094	2,569,267	3,159,472	4,069,988	+226.6
Kidney US											
North	54,741	51,882	52,878	56,697	59,085	38,525	48,170	63,237	74,035	83,973	+53.4
Northeast	172,199	177,627	188,235	210,149	212,291	135,877	183,296	227,522	265,923	331,789	+92.7
Southeast	503,938	513,675	544,401	563,510	591,578	422,830	515,732	575,068	640,178	699,522	+38.8
South	132,028	133,878	139,035	146,265	161,610	121,387	144,715	163,257	173,929	172,685	+30.8
Midwest	65,269	63,735	61,956	69,545	70,248	52,718	62,800	80,762	87,409	96,885	+48.4
Total	928,175	940,797	986,505	1,046,166	1,094,812	771,337	954,713	1,109,846	1,241,474	1,384,854	+49.2
Nephrology consultation											
North	119,096	116,578	128,782	137,552	113,724	103,042	110,830	152,914	190,074	205,089	+72.2
Northeast	207,000	225,990	441,690	505,634	542,138	511,599	603,500	742,637	1,212,544	1,314,516	+535.0
Southeast	558,169	552,009	569,690	607,858	646,899	482,461	546,933	703,194	1,334,670	1,667,207	+198.7
South	199,890	199,202	201,303	227,994	239,429	189,246	232,248	267,510	480,326	754,427	+277.4
Midwest	75,019	97,200	107,770	112,481	108,660	89,539	105,571	132,371	228,066	304,861	+306.4
Total	1,159,174	1,190,979	1,449,235	1,591,519	1,650,850	1,375,887	1,599,082	1,998,626	3,445,680	4,246,100	+266.3
Kidney biopsy											
North	77	106	100	107	144	132	135	155	237	245	+218.2
Northeast	564	710	925	909	1,146	718	882	757	917	581	+3.0
Southeast	1,463	1,564	1,493	1,441	1,612	1,290	1,365	1,393	1,673	1,821	+24.5
South	548	676	747	607	554	602	527	515	598	610	+11.3
Midwest	182	254	219	258	187	168	182	140	177	136	-25.3
Total	2,834	3,310	3,484	3,322	3,643	2,910	3,091	2,960	3,602	3,393	+19.7
Multidisc. CKD care											
North	0	25	241	165	169	33	0	46	195	200	+2.6
Northeast	0	0	0	0	38	44	1,056	2,272	3,588	5,513	+53.7
Southeast	0	0	83	918	2,164	2,383	5,429	9,521	16,566	20,314	+22.6
South	0	0	0	174	282	519	739	3,343	6,590	11,363	+72.4
Midwest	0	0	31	286	569	579	560	752	1,011	1,152	+13.9
Total	0	25	355	1,543	3,222	3,558	7,784	15,934	27,950	38,542	+37.9
AVF											
North	1,610	1,599	2,109	2,013	2,101	2,058	2,620	2,568	2,696	2,528	+57.0
Northeast	9,463	9,698	10,600	10,949	11,838	10,730	11,529	11,213	12,245	12,723	+34.4
Southeast	15,386	15,286	15,230	16,527	17,613	16,900	16,431	17,274	17,576	16,351	+6.3
South	5,326	5,384	5,399	5,597	5,965	5,746	5,211	5,578	5,510	5,411	+1.6
Midwest	2,645	2,560	2,676	2,900	2,933	2,895	2,605	2,904	2,944	3,321	+25.6
Total	34,430	34,527	36,014	37,986	40,450	38,329	38,396	39,537	40,971	40,334	+17.1
Kidney transplantation											
North	79	119	90	70	72	16	31	39	116	145	+83.5
Northeast	862	778	893	1,008	1,030	664	746	800	915	811	-5.9
Southeast	2,677	2,585	2,768	2,688	2,898	2,305	2,401	2,553	2,745	2,620	-2.1
South	1,189	1,275	1,371	1,413	1,353	1,017	882	1,109	1,246	1,238	+4.1
Midwest	118	175	190	223	268	240	196	165	280	251	+112.7
Total	4,925	4,932	5,312	5,402	5,621	4,242	4,256	4,666	5,302	5,065	+2.8

Abbreviations – CKD, chronic kidney disease. SUS, Brazilian Unified Health System. US, ultrasound. Multidisc. CKD care, multidisciplinary CKD care. AVF, arteriovenous fistula.

Notes – Rel. Δ (%), relative delta: relative variation between 2015 and 2024 for all variables, except multidisc. CKD care (Rel. Δ [%] between 2023 and 2024).

Between 2015 and 2024, the population undergoing chronic dialysis in the SUS increased from 93,085 to 121,914 (+31.0%); the greatest relative increase was in the North Region (+60.4%) and the smallest in the Southeast Region (+19.0%). The number of people undergoing HD increased from 86,755 to 116,658 (+34.5%) in the SUS; the largest relative increase was in the North Region (+70.8%) and the smallest in the Southeast Region (+22.0%). During the same period, the number of PD patients in the SUS decreased from 6,331 to 5,256 (−17.0%); the location with the highest relative decrease was the Northeast Region (−59.4%), while the Midwest Region showed the highest increase in the number of PD patients (+102.1%) (Table 3).

**Table 3:** Estimated number of patients on chronic dialysis funded by the SUS, by region in Brazil (2015–2024)

Procedure (n) Region	2015	2016	2017	2018	2019	2020	2021	2022	2023	2024	Rel. Δ (%)
HD											
North	4,008	4,185	4,810	5,135	5,477	5,790	5,868	6,270	6,482	6,848	+70.8
Northeast	22,595	23,443	24,308	25,732	27,321	28,511	28,991	30,551	32,711	35,254	+56.0
Southeast	41,718	42,660	43,057	44,419	45,858	47,004	46,417	47,121	49,228	50,895	+22.0
South	11,338	11,518	11,825	12,343	12,941	13,233	13,268	13,750	14,394	14,787	+30.4
Midwest	7,095	7,220	7,470	7,789	8,309	8,433	8,179	8,266	8,699	8,874	+25.1
Total	86,755	89,026	91,470	95,419	99,904	102,972	102,724	105,958	111,514	116,658	+34.5
PD											
North	358	298	226	204	190	165	161	151	166	154	-56.8
Northeast	1,097	952	770	641	579	534	506	459	477	446	-59.4
Southeast	3,473	3,434	3,313	3,236	3,243	3,278	3,086	2,896	2,893	2,862	-17.6
South	1,087	1,041	1,079	1,082	1,125	1,150	1,077	1,049	1,107	1,153	+6.1
Midwest	317	355	414	470	480	478	500	504	571	640	+102.1
Total	6,331	6,081	5,801	5,633	5,616	5,604	5,330	5,059	5,214	5,256	-17.0
HD + PD											
North	4,366	4,483	5,035	5,340	5,666	5,956	6,029	6,421	6,649	7,003	+60.4
Northeast	23,692	24,395	25,078	26,373	27,899	29,045	29,497	31,011	33,187	35,700	+50.7
Southeast	45,191	46,095	46,370	47,655	49,101	50,282	49,503	50,017	52,121	53,757	+19.0
South	12,425	12,559	12,904	13,425	14,065	14,383	14,345	14,799	15,501	15,941	+28.3
Midwest	7,412	7,575	7,884	8,259	8,788	8,911	8,679	8,770	9,270	9,514	+28.4
Total	93,085	95,107	97,272	101,052	105,520	108,577	108,054	111,018	116,728	121,914	+31.0

Abbreviations – SUS, Brazilian Unified Health System. HD, hemodialysis. PD, peritoneal dialysis.

Notes – Estimated number of prevalent chronic dialysis patients (HD and PD) funded by the SUS. Rel. Δ (%), relative delta: relative variation between 2024 and 2015.

**Figure 1:**
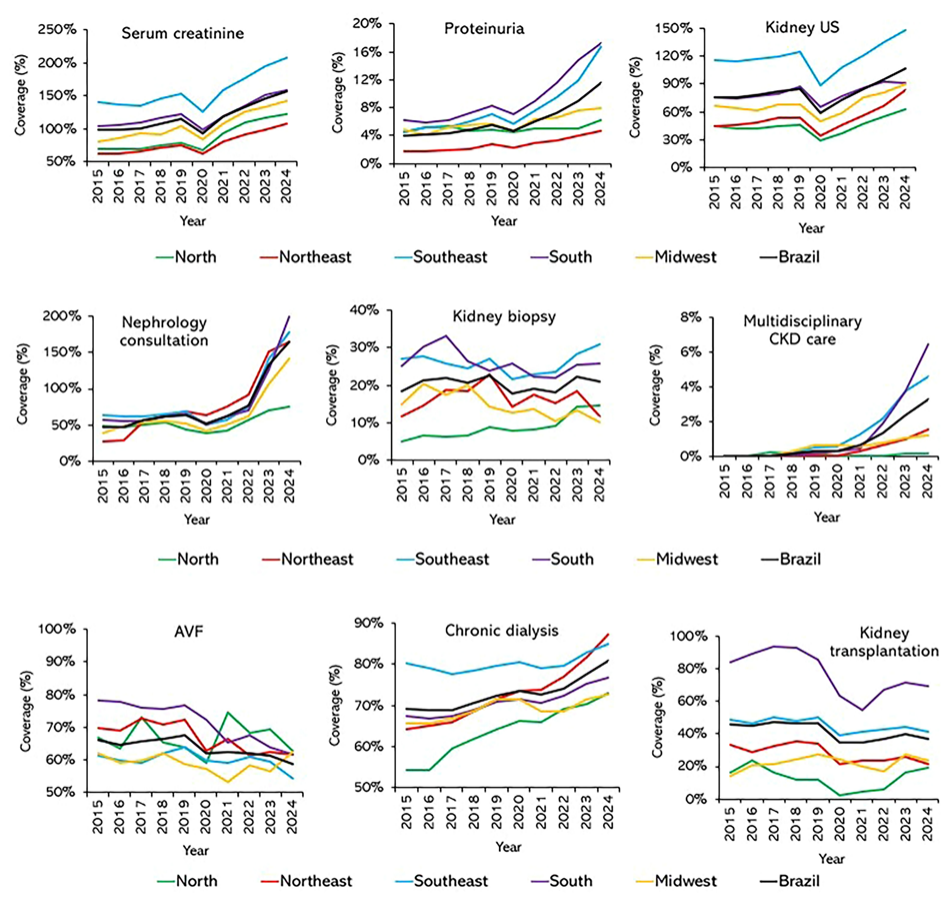
Coverage of procedures related to CKD care in the SUS, by region in Brazil (2015–2024).

Between 2014 and 2025, the coverage rate for serum creatinine testing increased from 69.6% to 121.8% in Brazil (an increase of 75.0%); the relative increase was highest in the South Region (+74.8%) and lowest in the Northeast Region (+46.7%). In the same period, proteinuria testing coverage increased from 3.9% to 11.5% in the country (an increase of 190.9%); this increase was greater in the Southeast Region (+266.1%) and lower in the North Region (+38.5%). The percentage of kidney ultrasound coverage increased from 76.1% to 107.1% in Brazil (an increase of 40.8%); the relative increase was higher in the Northeast Region (+86.9%) and lower in the South Region (+20.9%). Coverage of nephrology consultations increased from 47.5% to 164.3% between 2015 and 2024 (an increase of 245.6%); this increase was higher in the Northeast Region (+515.9%) and lower in the North Region (+57.0%). The coverage rate for kidney biopsies increased from 18.6% to 21.0% in the country (an increase of 13.0%); this increase was greater in the North Region (+190.1%), while the Midwest Region showed a 32.3% decrease in this indicator. In 2024, coverage of multidisciplinary follow-up for CKD reached 3.3% in Brazil, being higher in the South Region (6.4%), followed by the Southeast (4.6%), Northeast (1.5%), Midwest (1.2%), and North (0.2%) regions. The percentage of AVF coverage dropped from 66.1% to 58.7% in the country (a reduction of 11.3%); this decline was greater in the South (−21.2%), and only one region showed a positive value for this indicator (the Midwest, with +0.5%). Between 2015 and 2024, chronic dialysis coverage in Brazil increased from 69.1% to 80.8% (an increase of 16.9%); this increase was greater in the Northeast Region (+35.8%) and lower in the Southeast Region (+5.9%). During the same period, kidney transplant coverage fell from 45.6% to 36.9% (a reduction of 19.2%); this decline was higher in the Northeast Region (−36.2%), and two regions recorded an increase in this indicator: the Midwest (+67.1%) and the North (+18.8%) (Figure 1 and Table S5).

Between 2015 and 2024, the use of PD in the SUS decreased from 6.8% to 4.3% in Brazil. The following regions experienced the same downward trend: North (−73.2%), Northeast (−3.0%), Southeast (−30.7%), and South (−17.3%). The only region showing an increase in this indicator was the Midwest (+57.3%) (Table 4).

**Table 4:** Percentage of peritoneal dialysis use in the SUS, by region in Brazil (2015–2024)

Region (%)	2015	2016	2017	2018	2019	2020	2021	2022	2023	2024	Rel. Δ (%)
North	8.2	6.6	4.5	3.8	3.4	2.8	2.7	2.4	2.5	2.2	-73.2
Northeast	4.6	3.9	3.1	2.4	2.1	1.8	1.7	1.5	1.4	1.2	-73.0
Southeast	7.7	7.4	7.1	6.8	6.6	6.5	6.2	5.8	5.6	5.3	-30.7
South	8.7	8.3	8.4	8.1	8.0	8.0	7.5	7.1	7.1	7.2	-17.3
Midwest	4.3	4.7	5.3	5.7	5.5	5.4	5.8	5.7	6.2	6.7	+57.3
Total	6.8	6.4	6.0	5.6	5.3	5.2	4.9	4.6	4.5	4.3	-36.6

Abbreviations – SUS: Brazilian Unified Health System; PD: peritoneal dialysis; HD: hemodialysis.

Notes – Use of peritoneal dialysis (%) = number of patients on PD × 100/number of patients on chronic dialysis (HD + PD). Rel. Δ (%), relative delta: relative variation between 2024 and 2015.

## Discussion

This study showed that between 2015 and 2024, there was a substantial increase in the coverage of serum creatinine testing, kidney ultrasounds, nephrology consultations, and chronic dialysis in the SUS. In 2024, there were still significant deficits in the coverage of proteinuria testing, multidisciplinary care of pre-dialysis CKD, kidney biopsy, arteriovenous fistula, and kidney transplantation. The North, Midwest, and Northeast regions, compared to the Southeast and South regions, had greater shortages in nearly all evaluated procedures.

### Advances in the SUS

The SUS is one of the most comprehensive public health policies worldwide^16^. Many SUS initiatives are recognized by the World Health Organization as examples for other countries, such as the Family Health Program, the National Immunization Program, and access to antiretroviral therapy for human immunodeficiency virus infection, among others^17,18,19^. Regarding CKD care, Brazil is one of the few countries to provide universal coverage for chronic dialysis and kidney transplantation^20^. Nevertheless, Brazilian studies have shown that important gaps remain in the comprehensive care of individuals at risk for or living with CKD^21,22,23^.

### Regional Differences

The regional differences in CKD care coverage observed in this study are likely influenced by the social, economic, demographic, and epidemiological conditions prevailing in each region of the country^24^. Advanced age, HTN, DM, and obesity are known to be risk factors for CKD^1,9,25^. The percentage of elderly people in the North, Midwest, and Northeast regions is lower than in the Southeast and South^6^, which would imply lower healthcare demand from this population and, therefore, lower calculated coverage results. When considering only the state capitals of Brazil, the prevalence of obesity and DM shows less regional variation, while the prevalence of HTN is generally higher in the Southeast and South^5^. Unfortunately, it was not possible to adjust our coverage findings by age and comorbidity profile. However, factors such as income, education, basic sanitation, housing, and transportation, among others, are determinants of health and healthcare access that are as important as – or even more important than – age and comorbidity profile^26^.

### Ancillary Exams

This study demonstrated that the proteinuria screening coverage in Brazil (combined 24-hour albuminuria and proteinuria measurements) is only 12%, a result corroborated by previous studies^21,22,23,27^. Overall, the main barrier to improving this indicator is the lack of knowledge on the importance of this test among healthcare professionals and the general population^1,3,28^. In Brazil, this is a low-cost, widely available test, and its use in at-risk individuals has been recommended in ministerial guidelines for over a decade^9,13,29^. This analysis demonstrated coverage rates for serum creatinine and kidney ultrasounds exceeding 100% in some regions, which could be partly explained by repeated tests on the same individual. It is possible that this apparent excess of serum creatinine and kidney ultrasound tests is due to a lack of integration between information systems and the fragmentation of healthcare within the SUS^30^. Another possible explanation is the performance of unnecessary tests on patients without risk factors for CKD; indeed, awareness of the early stages of CKD among healthcare professionals and the general population is lower than 20% and 5%, respectively^1,3,28^.

The low coverage of kidney biopsies observed in this study is possibly explained by inadequate remuneration and insufficient availability, as this procedure requires physical and human resources that are often unavailable in many regions of Brazil (immunofluorescence and electron microscopy techniques, physicians specializing in kidney pathology, among others)^10,13^. Considering that kidney biopsies are requested by nephrologists, late referral to this professional contributes to fewer requests for this test, as kidney biopsies are not indicated for advanced cases of CKD^29,31^.

### Nephrology Consultations

It is plausible that the substantial increase in coverage for nephrology consultations, evident after 2022, is partly attributable to the authorization, granted by certain state authorities, for chronic dialysis facilities to invoice the SUS for a medical consultation each time a hemodialysis session is performed^32^. As a result, this indicator would no longer reflect outpatient consultations for SUS patients who are not on dialysis. In addition, the apparent excess of appointments might be explained by the referral of patients who could otherwise be monitored solely in primary healthcare services^1,23^. Although nephrologist density has increased across all regions of Brazil (number of professionals per 100,000 inhabitants), the percentage of these professionals working in the SUS and in teaching institutions has declined^33^. This trend suggests an increase in the number of nephrologists working in healthcare services that serve people with private health insurance (health insurance plans and/or out-of-pocket payment) (Table S6).

### Multidisciplinary CKD Care

Multidisciplinary care procedures for pre-dialysis CKD were established by the Brazilian Ministry of Health in 2014 with the aim of encouraging dialysis clinics to provide care for this group of patients, given the availability of specialized professionals in these settings^29^. However, federal funding for this initiative has been insufficient^10^, and only a few states, which have opted for local incentives, have achieved increased coverage of these procedures^34,35^.

### Kidney Replacement Therapy (KRT)

The estimated dialysis population in this study is lower than the figures reported in Brazilian dialysis surveys conducted by the Brazilian Society of Nephrology (SBN, in Portuguese)^36^. The main explanation is that our study considered only procedures performed in the SUS, while the SBN surveys included individuals undergoing dialysis in both the SUS and supplementary health systems. Furthermore, this study was based on KRT procedures performed only in services accredited by the Brazilian Ministry of Health, although there may be others funded exclusively by state or local resources. The parameter used to determine the need for chronic dialysis considered prevalent dialysis patients, thus not distinguishing these individuals from those who had recently initiated KRT.

The findings of this study demonstrated that, in Brazil, chronic dialysis coverage in 2024 exceeded that of kidney transplantation by more than twofold. Over the past decade, the provision of dialysis care has shown an upward trend in all regions of Brazil, in contrast to the decline or stagnation in kidney transplant coverage. It should be noted that regional inequalities in chronic dialysis have been progressively corrected, yet disparities in kidney transplant coverage among the country’s regions remain significant^37^.

Over the last decade, the increase in chronic dialysis coverage in Brazil has been exclusively due to the rise in the prevalence of individuals on HD. This result coincided temporally with the opening of the Brazilian healthcare sector to foreign capital investment^38^. Unlike kidney transplantation, dialysis services in Brazil are primarily provided by private, for-profit companies^8,36,39^. The use of PD has declined in all regions except the Midwest, owing to local incentives^40^. Possible explanations include underfunding at the federal level, greater profitability of HD compared to PD for service providers, the absence of equipment and supply manufacturing centers distributed across different regions of the country, excessive logistical costs, and a lack of qualified services and trained professionals, among other factors^39^.

### Limitations

The limitations of this study should be acknowledged. First, the results cannot be generalized to individuals with supplementary health insurance, who currently represent about a quarter of the Brazilian population. Second, the use of the SUS by people with supplementary health insurance was considered only for KRT procedures (dialysis and kidney transplantation), and it was not possible to obtain information on the number of people with private health insurance who used the SUS for low- and medium-complexity procedures (exams and consultations). Third, the information systems did not allow us to stratify supplementary health coverage and the number of procedures according to age group and comorbidity profile. Fourth, the variation in need parameters over time was not considered. Finally, the methodology employed did not enable us to assess potential duplication of procedures performed on the same individual.

## Conclusions

This study essentially used tools that are part of the work routine of SUS administrators. It was possible to identify and quantify several shortcomings in public and comprehensive care for CKD. While recognizing the demand for new technological resources and for the improvement and integration of SUS databases, the information currently available may guide actions aimed at expanding the service network and correcting the discrepancies observed.

## Data Availability

The data supporting this article are available at reasonable request to the corresponding author.

## Supplementary Material

Table S1 – SIGTAP codes for procedures related to chronic kidney disease care referred to in this study.

Table S2 – Number of hemodialysis sessions and kidney transplantations performed in the SUS among supplementary health insurance beneficiaries (2013–2022).

Table S3 – Formulas for calculating estimates of individuals undergoing chronic dialysis, based on the number of procedures performed.

Table S4 – Demographic data and supplementary health coverage, by region in Brazil (2015–2024).

Table S5 – Coverage of procedures related to CKD care in the SUS, by region in Brazil (2015–2024).

Table S6 – Nephrologist density and percentages of services provided by these professionals in the SUS and in teaching institutions, by region in Brazil (2015–2024).
