# Supplementary material for: Coverage of procedures related to chronic kidney disease care in the Brazilian Unified Health System (SUS): analysis of the 2015–2024 decade
Source: J Bras Nefrol. 2026 Jan 23;48(2):e20250144. doi: 10.1590/2175-8239-JBN-2025-0144en (PMC12893125; doi:10.1590/2175-8239-JBN-2025-0144en)
Supplement: Supplementary file 1 [file 2175-8239-jbn-48-2-e20250144-Table-S1.pdf]

Braz. J. Nephrol.
https://doi.org/10.1590/2175-8239-JBN-2025-0144pt

Material Suplementar para "Cobertura de procedimentos relacionados à assistência à Doença Renal Crônica no Sistema Único de Saúde do Brasil: análise da década 2015-2024"

Tabela S1 - Códigos SIGTAP dos procedimentos relacionados com a assistência à doença renal crônica e consultados neste estudo[1].

Procedimento	Código	Fonte de dados
Dosagem de creatinina	02.02.01.031-7	SIA
Dosagem de microalbumina na urina	02.02.05.009-2	SIA
Dosagem de proteínas (urina de 24 horas)	02.02.05.011-4	SIA
Ultrassonografia de aparelho urinário	02.05.02.005-4	SIA
Consulta médica especializada (CBO médico nefrologista)	03.01.01.007-2	SIA
Teleconsulta médica na atenção especializada (CBO médico nefrologista)	03.01.01.030-7	SIA
Biópsia de rim por punção	02.01.01.043-7	SIA
Biópsia de rim por punção	02.01.01.043-7	SIH
Acompanhamento multiprofissional DRC estágio 4	03.01.13.005-1	SIA
Acompanhamento multiprofissional DRC estágio 5 pré-diálise	03.01.13.006-0	SIA
Confecção de fistula arteriovenosa para acesso	04.06.02.008-6	SIA
Confecção de fistula arteriovenosa com enxerto de PTFE	04.18.01.001-3	SIA
Confecção de fistula arteriovenosa com enxerto autólogo	04.18.01.002-1	SIA
Confecção de fistula arteriovenosa para hemodiálise	04.18.01.003-0	SIA
Hemodiálise (máximo 3 sessões por semana)	03.05.01.010-7	SIA
Hemodiálise (1 sessão por semana - excepcionalidade)	03.05.01.009-3	SIA
Hemodiálise sorologia HIV e/ou HBV e/ou HCV (máximo 3 sessões por semana)	03.05.01.011-5	SIA
Hemodiálise sorologia HIV e/ou HBV e/ou HCV	03.05.01.012-3	SIA

Procedimento	Código	Fonte de dados
(1 sessão por semana - excepcionalidade)		
Hemodiálise pediátrica (máximo 4 sessões por semana)	03.05.01.020-4	SIA
Conjunto troca para DPA (paciente-mês)	07.02.10.004-8	SIA
Conjunto troca para DPAC (paciente-mês)	07.02.10.006-4	SIA
Conjunto troca para DPA (paciente-15 dias)	07.02.10.005-6	SIA
Conjunto troca para DPAC (paciente-15 dias)	07.02.10.008-0	SIA
Diálise peritoneal intermitente (máximo 2 sessões por semana)	03.05.01.002-6	SIA
Diálise peritoneal intermitente (1 sessão por semana - excepcionalidade)	03.05.01.001-8	SIA
Transplante de rim (órgão de doador vivo)	05.05.02.010-6	SIH
Transplante de rim (órgão de doador falecido)	05.05.02.009-2	SIH
Transplante simultâneo de pâncreas e rim	05.05.02.011-4	SIH

SIGTAP, Sistema de Gerenciamento da Tabela de Procedimentos, Medicamentos, Órteses, Próteses e Materiais Especiais do Sistema Único de Saúde (SUS). SIA, Sistema de Informações Ambulatoriais do SUS. CBO, código brasileiro de ocupações. SIH, Sistema de Informações Hospitalares do SUS. DRC, doença renal crônica. PTFE, politetrafluoretileno. HIV, vírus da imunodeficiência humana. HBV, vírus da hepatite B. HCV, vírus da hepatite C. DPA, diálise peritoneal automática. DPAC, diálise peritoneal ambulatorial contínua.

Referências

1. Brasil. Ministério da Saúde. DATASUS Tecnologia da Informação a Serviço do SUS. Sistema de Gerenciamento da Tabela de Procedimentos, Medicamentos e OPM (Órteses, próteses e materiais) do SUS (SIGTAP) [Internet]. 2025 [citado em 2025 maio 4]. Disponível em: http://sigtap.datasus.gov.br/tabela-unificada/app/sec/inicio.jsp.
