# Supplementary material for: Coverage of procedures related to chronic kidney disease care in the Brazilian Unified Health System (SUS): analysis of the 2015–2024 decade
Source: J Bras Nefrol. 2026 Jan 23;48(2):e20250144. doi: 10.1590/2175-8239-JBN-2025-0144en (PMC12893125; doi:10.1590/2175-8239-JBN-2025-0144en)
Supplement: Supplementary file 2 [file 2175-8239-jbn-48-2-e20250144-Table-S2.pdf]

Braz. J. Nephrol.
https://doi.org/10.1590/2175-8239-JBN-2025-0144pt

Material Suplementar para "Cobertura de procedimentos relacionados à assistência à Doença Renal Crônica no Sistema Único de Saúde do Brasil: análise da década 2015-2024"

Tabela S2 - Número de sessões de hemodiálise e de transplantes renais realizados no SUS por beneficiários de saúde suplementar (2013-2022)[1].

**Variável Região**	**2013**	**2022**	**2015**	**2016**	**2017**	**2018**	**2019**	**2020**	**2021**	**2022**
Sessões de HD (n)										
Norte	32.500	314.574	26.156	32.630	35.581	32.578	29.263	28.301	28.366	28.704
Nordeste	143.559	148.746	118.989	146.198	139.022	129.558	121.758	116.584	115.726	126.399
Sudeste	623.376	562.679	544.271	602.706	556.855	516.594	488.423	478.101	464.555	474.968
Sul	69.160	101.413	81.250	96.811	92.339	89.414	84.097	78.962	81.146	83.876
Centro-oeste	41.665	44.265	31.538	39.364	40.352	39.793	37.596	39.520	41.743	45.942
Total	910.260	1.171.677	802.204	917.709	864.149	807.937	761.137	741.468	731.536	759.889
Pacientes em HD (n)										
Norte	208	2.017	168	209	228	209	188	181	182	184
Nordeste	920	954	763	937	891	831	781	747	742	810
Sudeste	3.996	3.607	3.489	3.864	3.570	3.312	3.131	3.065	2.978	3.045
Sul	443	650	521	621	592	573	539	506	520	538
Centro-oeste	267	284	202	252	259	255	241	253	268	295
Total	5.835	7.511	5.142	5.883	5.539	5.179	4.879	4.753	4.689	4.871
TX renal (n)										
Norte	14	9	4	17	7	4	6	1	2	1

Variável Região	2013	2022	2015	2016	2017	2018	2019	2020	2021	2022
Nordeste	123	124	132	101	111	110	11	3	6	12
Sudeste	580	582	518	470	498	375	169	33	67	71
Sul	193	161	155	174	180	173	75	5	6	13
Centro-oeste	38	20	12	17	25	26	20	3	4	11
Total	948	896	821	779	821	688	281	45	85	108

SUS, Sistema Único de Saúde. SS, saúde suplementar. HD, hemodiálise. TX renal, transplante renal. A estimativa de pacientes da SS em HD no SUS foi calculada a partir do número de sessões desse procedimento.

Referências

1. Brasil. Ministério da Saúde. Agência Nacional de Saúde Suplementar. Panorama do Ressarcimento ao SUS. Dezembro de 2024. Dados e informações [Internet]. 2024 [citado em 2025 maio 4]. Disponível em: https://www.gov.br/ans/pt-br/acesso-a-informacao/perfil-do-setor/dados-e-indicadores-do-setor.
