# Supplementary material for: Coverage of procedures related to chronic kidney disease care in the Brazilian Unified Health System (SUS): analysis of the 2015–2024 decade
Source: J Bras Nefrol. 2026 Jan 23;48(2):e20250144. doi: 10.1590/2175-8239-JBN-2025-0144en (PMC12893125; doi:10.1590/2175-8239-JBN-2025-0144en)
Supplement: Supplementary file 3 [file 2175-8239-jbn-48-2-e20250144-Table-S3.pdf]

Braz. J. Nephrol.
https://doi.org/10.1590/2175-8239-JBN-2025-0144pt

Material Suplementar para "Cobertura de procedimentos relacionados à assistência à Doença Renal Crônica no Sistema Único de Saúde do Brasil: análise da década 2015-2024"

Tabela S3 - Fórmulas de cálculo das estimativas de pessoas em diálise crônica, a partir do montante de procedimentos realizados[1].

Método	Procedimento	Estimativa da média de pacientes prevalentes no ano*
HD	Hemodiálise (máximo 3 sessões por semana)	Σ de sessões / 13 / 12
	Hemodiálise (1 sessão por semana - excepcionalidade)	
	Hemodiálise sorologia HIV e/ou HBV e/ou HCV (máximo 3 sessões por semana)	
	Hemodiálise sorologia HIV e/ou HBV e/ou HCV (1 sessão por semana - excepcionalidade)	
	Hemodiálise pediátrica (máximo 4 sessões por semana)	Σ de sessões / 16 / 12
DP	Conjunto troca para DPA (paciente-mês)	Σ de procedimentos / 1 / 12
	Conjunto troca para DPAC (paciente-mês)	Σ de procedimentos / 1 / 12
	Conjunto troca para DPA (paciente-15 dias)	Σ de procedimentos / 2 / 12
	Conjunto troca para DPAC (paciente-15 dias)	Σ de procedimentos / 2 / 12
	DPI (máximo 2 sessões por semana)	Σ de sessões / 8 / 12

*Todos os cálculos se baseiam na somatória (Σ) de procedimentos aprovados pelo SUS por ano e informados no Sistema de Informações Ambulatoriais (SIA-SUS). Em cada célula da terceira coluna da tabela, o primeiro número da divisão corresponde à quantidade média estimada de procedimentos realizados por mês e o segundo número, à quantidade de meses do período avaliado (1 ano). SUS, Sistema Único de Saúde. HD, hemodiálise. DP, diálise peritoneal. HIV, vírus da imunodeficiência humana. HBV, vírus da hepatite B. HCV, vírus da hepatite C. DPA, diálise peritoneal automática. DPAC, diálise peritoneal ambulatorial contínua. DPI, diálise peritoneal intermitente.

Referências

1. Brasil. Ministério da Saúde. DATASUS Tecnologia da Informação a Serviço do SUS. Sistema de Gerenciamento da Tabela de Procedimentos, Medicamentos e OPM (Órteses, próteses e materiais) do SUS (SIGTAP) [Internet]. 2025 [citado em 2025 maio 4]. Disponível em: http://sigtap.datasus.gov.br/tabela-unificada/app/sec/inicio.jsp
