# Supplementary material for: Coverage of procedures related to chronic kidney disease care in the Brazilian Unified Health System (SUS): analysis of the 2015–2024 decade
Source: J Bras Nefrol. 2026 Jan 23;48(2):e20250144. doi: 10.1590/2175-8239-JBN-2025-0144en (PMC12893125; doi:10.1590/2175-8239-JBN-2025-0144en)
Supplement: Supplementary file 4 [file 2175-8239-jbn-48-2-e20250144-Table-S4.pdf]

Braz. J. Nephrol.
https://doi.org/10.1590/2175-8239-JBN-2025-0144pt

Material Suplementar para "Cobertura de procedimentos relacionados à assistência à Doença Renal Crônica no Sistema Único de Saúde do Brasil: análise da década 2015-2024"

Tabela S4 - Dados demográficos e cobertura de saúde suplementar, segundo regiões do Brasil (2015-2024).

Variável Região	2015	2016	2017	2018	2019	2020	2021	2022	2023	2024	Δ Rel. (%)
População (n)											
Norte	17.236.948	17.429.371	17.606.346	17.783.329	17.970.930	18.137.222	18.272.491	18.403.491	18.535.113	18.669.345	+8,3
Nordeste	55.204.481	55.483.846	55.729.786	55.987.936	56.272.587	56.524.887	56.713.897	56.848.401	56.970.423	57.112.096	+3,5
Sudeste	85.494.023	86.032.875	86.509.018	86.949.467	87.386.818	87.778.972	88.016.928	88.178.052	88.387.852	88.617.693	+3,7
Sul	29.062.489	29.316.913	29.561.726	29.807.638	30.066.296	30.323.248	30.529.735	30.704.015	30.903.366	31.113.021	+7,1
Centro-oeste	15.405.701	15.608.920	15.804.681	16.000.668	16.203.468	16.400.560	16.570.591	16.729.024	16.898.404	17.071.595	+10,8
Total	202.403.642	203.871.925	205.211.557	206.529.038	207.900.099	209.164.889	210.103.642	210.862.983	211.695.158	212.583.750	+5,0
Pop. ≥ 20 anos (n)											
Norte	10.520.961	10.754.772	10.984.528	11.212.498	11.449.172	11.673.796	11.869.427	12.064.408	12.269.093	12.476.426	+18,6
Nordeste	36.841.903	37.363.737	37.870.217	38.363.135	38.870.304	39.361.128	39.789.676	40.183.683	40.582.316	40.991.588	+11,3
Sudeste	61.111.843	61.859.497	62.572.266	63.244.804	63.922.076	64.568.227	65.073.304	65.520.377	66.014.310	66.519.843	+8,8
Sul	20.828.047	21.135.516	21.435.000	21.728.696	22.031.050	22.330.897	22.581.698	22.802.779	23.043.786	23.292.123	+11,8

Variável Região	2015	2016	2017	2018	2019	2020	2021	2022	2023	2024	Δ Rel. (%)
Centro-oeste	10.515.727	10.726.792	10.933.879	11.136.329	11.343.907	11.549.077	11.730.893	11.905.013	12.089.748	12.277.428	+16,8
Total	139.818.481	141.840.314	143.795.890	145.685.462	147.616.509	149.483.125	151.044.998	152.476.260	153.999.253	155.557.408	+11,3
Pop. ≥ 60 anos (n)											
Norte	1.360.593	1.424.371	1.491.444	1.561.723	1.634.716	1.701.969	1.761.235	1.828.263	1.908.027	1.992.741	+46,5
Nordeste	6.380.848	6.579.657	6.786.474	7.004.192	7.233.983	7.459.032	7.673.443	7.905.831	8.173.561	8.467.135	+32,7
Sudeste	11.583.471	12.034.039	12.500.599	12.980.463	13.468.867	13.935.387	14.341.669	14.757.419	15.241.645	15.747.890	+36,0
Sul	4.018.702	4.183.898	4.354.776	4.531.798	4.715.523	4.900.765	5.067.640	5.231.620	5.420.769	5.622.774	+39,9
Centro-oeste	1.574.004	1.649.423	1.728.597	1.811.342	1.897.672	1.982.290	2.059.130	2.141.366	2.237.489	2.339.077	+48,6
Total	24.917.618	25.871.388	26.861.890	27.889.518	28.950.761	29.979.443	30.903.117	31.864.499	32.981.491	34.169.617	+37,1
Cobertura SS (%)											
Norte	10,8	10,1	9,8	9,4	9,3	9,2	9,4	9,5	9,5	9,7	-10,5
Nordeste	12,1	12,0	11,7	11,6	11,6	11,5	11,5	11,8	12,1	12,4	+2,4
Sudeste	36,4	34,6	33,2	32,6	32,2	32,1	32,4	33,0	33,4	33,6	-7,8
Sul	24,6	23,9	23,5	23,3	22,9	22,7	22,6	23,1	23,5	23,8	-3,2
Centro-oeste	20,7	20,5	20,1	19,6	19,8	19,9	20,2	20,3	20,7	21,0	+1,4
Total	24,7	23,7	22,9	22,5	22,3	22,2	22,4	22,8	23,1	24,0	-2,8

Δ Rel. (%), delta relativo: variação relativa entre 2024 e 2015. SS, saúde suplementar.
