# Supplementary material for: Coverage of procedures related to chronic kidney disease care in the Brazilian Unified Health System (SUS): analysis of the 2015–2024 decade
Source: J Bras Nefrol. 2026 Jan 23;48(2):e20250144. doi: 10.1590/2175-8239-JBN-2025-0144en (PMC12893125; doi:10.1590/2175-8239-JBN-2025-0144en)
Supplement: Supplementary file 5 [file 2175-8239-jbn-48-2-e20250144-Table-S5.pdf]

Braz. J. Nephrol.
https://doi.org/10.1590/2175-8239-JBN-2025-0144pt

Material Suplementar para "Cobertura de procedimentos relacionados à assistência à Doença Renal Crônica no Sistema Único de Saúde do Brasil: análise da década 2015-2024"

Tabela S5 - Cobertura de procedimentos relacionados com a assistência à DRC no SUS, segundo regiões do Brasil (2015-2024)[1,2].

**Cobertura (%)**<br>**Região**	**2015**	**2016**	**2017**	**2018**	**2019**	**2020**	**2021**	**2022**	**2023**	**2024**	**Δ Rel. (%)**
Creatinina	69,6	69,1	69,3	74,8	79,2	68,3	93,7	108,9	116,1	121,8	+75,0
Norte	61,6	61,2	65,6	71,7	75,7	61,8	81,0	90,5	98,6	106,8	+73,4
Nordeste	141,2	137,6	134,6	146,3	153,3	126,2	157,7	176,0	195,2	207,1	+46,7
Sudeste	104,3	105,2	110,2	116,3	123,0	98,3	117,6	135,5	150,9	159,4	+52,8
Sul	81,1	85,3	92,4	91,4	104,5	83,2	108,3	125,3	133,4	141,8	+74,8
Centro-oeste	99,2	98,6	100,3	108,0	114,3	93,5	118,2	133,2	146,3	157,0	+58,2
Total	69,6	69,1	69,3	74,8	79,2	68,3	93,7	108,9	116,1	121,8	+75,0
Proteinúria											
Norte	4,5	5,2	5,3	4,6	4,8	4,6	5,1	5,1	5,1	6,2	+38,5
Nordeste	1,8	1,8	1,9	2,2	2,7	2,3	3,0	3,4	4,0	4,6	+151,8
Sudeste	4,6	5,2	5,2	6,1	7,1	5,8	7,6	9,5	11,9	16,7	+266,1
Sul	6,1	5,9	6,2	7,2	8,3	7,1	9,0	11,5	14,7	17,2	+180,1
Centro-oeste	4,8	4,1	5,2	5,5	5,7	4,6	6,5	6,6	7,6	7,9	+64,7
Total	3,9	4,2	4,3	4,9	5,6	4,7	6,1	7,3	8,9	11,5	+190,9
US renal											
Norte	44,5	41,4	41,6	44,0	45,3	29,2	36,4	47,4	55,2	62,2	+39,9
Nordeste	44,4	45,5	47,8	53,1	53,3	34,0	45,7	56,7	66,4	82,9	+86,9
Sudeste	115,9	114,1	117,7	120,2	124,8	88,7	108,3	121,7	136,0	148,6	+28,2
Sul	75,3	75,0	76,8	79,9	87,1	64,7	76,6	86,5	92,0	91,0	+20,9
Centro-oeste	66,8	64,2	61,3	67,6	67,5	50,2	59,4	75,7	81,5	89,8	+34,4
Total	76,1	75,6	77,9	81,7	84,7	59,2	73,2	85,2	95,4	107,1	+40,8
Consulta com nefrologista											
Norte	48,4	46,5	50,7	53,4	43,6	39,1	41,9	57,4	70,8	76,0	+57,0

Cobertura (%) Região	2015	2016	2017	2018	2019	2020	2021	2022	2023	2024	Δ Rel. (%)
Nordeste	26,7	28,9	56,1	63,9	68,1	63,9	75,2	92,6	151,4	164,2	+515,9
Sudeste	64,2	61,3	61,6	64,8	68,2	50,6	57,4	74,4	141,8	177,1	+175,9
Sul	57,0	55,8	55,6	62,3	64,5	50,4	61,5	70,9	127,0	198,9	+248,9
Centro-oeste	38,4	49,0	53,3	54,6	52,2	42,6	49,9	62,1	106,4	141,3	+268,0
Total	47,5	47,8	57,2	62,2	63,9	52,8	61,3	76,7	132,4	164,3	+245,6
Biópsia renal											
Norte	5,0	6,8	6,3	6,6	8,8	8,0	8,2	9,3	14,1	14,5	+190,1
Nordeste	11,6	14,5	18,8	18,4	23,0	14,4	17,6	15,1	18,3	11,6	-0,1
Sudeste	26,9	27,8	25,8	24,6	27,2	21,6	22,9	23,6	28,4	31,0	+15,0
Sul	25,0	30,3	33,0	26,5	23,9	25,7	22,3	21,8	25,3	25,7	+2,9
Centro-oeste	14,9	20,5	17,3	20,1	14,4	12,8	13,8	10,5	13,2	10,1	-32,3
Total	18,6	21,3	22,0	20,8	22,6	17,9	18,9	18,2	22,1	21,0	+13,0
Ac. multip. DRC											
Norte	0,0	0,0	0,2	0,2	0,2	0,0	0,0	0,0	0,2	0,2	+1,1
Nordeste	0,0	0,0	0,0	0,0	0,0	0,0	0,3	0,6	1,0	1,5	+52,6
Sudeste	0,0	0,0	0,0	0,2	0,5	0,5	1,2	2,2	3,8	4,6	+22,0
Sul	0,0	0,0	0,0	0,1	0,2	0,3	0,4	1,9	3,7	6,4	+71,2
Centro-oeste	0,0	0,0	0,0	0,3	0,6	0,6	0,6	0,8	1,1	1,2	+12,6
Total	0,0	0,0	0,0	0,1	0,3	0,3	0,7	1,4	2,4	3,3	+38,1
FAV											
Norte	66,9	63,7	73,1	65,3	63,9	59,2	74,4	68,3	69,3	62,9	-6,0
Nordeste	69,8	68,9	72,7	70,9	72,2	62,7	66,3	61,2	62,4	61,7	-11,6
Sudeste	61,5	59,7	59,0	62,0	64,0	59,9	59,0	61,1	59,5	54,4	-11,4
Sul	78,3	77,9	76,1	75,6	76,8	72,4	65,5	67,6	63,8	61,7	-21,2
Centro-oeste	62,1	59,1	59,7	62,1	58,8	57,2	53,1	58,6	56,4	62,4	+0,5
Total	66,1	64,6	65,6	66,3	67,5	62,0	62,3	62,2	61,2	58,7	-11,3
Diálise crônica											
Norte	54,2	54,3	59,6	61,9	64,2	66,2	66,0	69,1	70,4	73,0	+34,6
Nordeste	64,3	65,2	66,0	68,5	71,4	73,4	73,8	77,0	81,8	87,3	+35,8
Sudeste	80,3	79,2	77,6	78,4	79,6	80,6	79,0	79,8	82,9	85,0	+5,9
Sul	67,5	66,8	67,4	69,0	71,0	71,5	70,5	72,4	75,3	76,8	+13,8
Centro-oeste	65,8	65,8	66,9	68,5	71,6	71,4	68,7	68,5	71,6	72,6	+10,3

Cobertura (%) Região	2015	2016	2017	2018	2019	2020	2021	2022	2023	2024	Δ Rel. (%)
Total	69,1	68,9	69,0	70,5	72,5	73,6	72,6	74,2	77,5	80,8	+16,9
TX renal											
Norte	16,1	23,9	16,3	12,0	11,7	2,5	4,7	5,6	16,1	19,2	+18,8
Nordeste	33,2	29,2	32,7	35,2	34,2	21,2	23,5	24,0	25,7	21,2	-36,2
Sudeste	49,2	47,0	50,3	47,8	50,3	39,1	41,2	43,3	44,8	41,5	-15,5
Sul	84,2	89,6	94,1	93,6	86,0	63,4	55,0	67,0	72,1	69,7	-17,3
Centro-oeste	14,2	20,6	21,6	24,4	27,6	24,4	20,3	16,9	27,2	23,7	+67,1
Total	45,6	45,0	47,7	46,9	47,0	34,5	34,7	37,1	40,2	36,9	-19,2

Elaborado a partir do montante de procedimentos realizados no SUS e de parâmetros de necessidade do Ministerio da Saúde[1,2]. Δ Rel. (%), delta relativo: variação relativa entre 2015 e 2024 para todas as variáveis, exceto ac. multip. DRC (2023 e 2024). DRC, doença renal crônica. SUS, Sistema Único de Saúde. US, ultrassonografia. DRC, doença renal crônica. Ac. Multip. DRC, acompanhamento multiprofissional da DRC. FAV, fístula arteriovenosa. TX, transplante renal.

Referências

1. Brasil. Ministério da Saúde. DATASUS Tecnologia da Informação a Serviço do SUS. Sistema de Informações Ambulatorial e Hospitalares [Internet]. 2025 [citado em 2025 maio 4]. Disponível em: https://datasus.saude.gov.br/informacoes-de-saude-tabnet.

2. Brasil. Ministério da Saúde. Secretaria de Atenção à Saúde. Departamento de Regulação, Avaliação e Controle. Critérios e parâmetros assistenciais para o planejamento e programação de ações e serviços de saúde no âmbito do Sistema Único de Saúde [Internet]. Brasília: Ministério da Saúde; 2017 [citado em 2025 maio 4]. Disponível em: https://www.gov.br/saude/pt-br/acesso-a-informacao/gestao-do-sus/programacao-regulacao-controle-e-financiamento-da-mac/programacao-assistencial/arquivos/caderno-1-criterios-e-parametros-assistenciais-1-revisao.pdf.
