# Supplementary material for: Coverage of procedures related to chronic kidney disease care in the Brazilian Unified Health System (SUS): analysis of the 2015–2024 decade
Source: J Bras Nefrol. 2026 Jan 23;48(2):e20250144. doi: 10.1590/2175-8239-JBN-2025-0144en (PMC12893125; doi:10.1590/2175-8239-JBN-2025-0144en)
Supplement: Supplementary file 6 [file 2175-8239-jbn-48-2-e20250144-Table-S6.pdf]

https://doi.org/10.1590/2175-8239-JBN-2025-0144pt

Material Suplementar para "Cobertura de procedimentos relacionados à assistência à Doença Renal Crônica no Sistema Único de Saúde do Brasil: análise da década 2015-2024"

Tabela S6 - Densidade de nefrologistas e percentuais de atendimento desses profissionais no SUS e em instituições de ensino, segundo regiões do Brasil (2015-2024)[1].

Variável Região	2015	2016	2017	2018	2019	2020	2021	2022	2023	2024	Δ Rel. (%)
Densidade de nefrologistas (por 100.000 hab.)											
Norte	0,9	1,0	1,0	1,1	1,2	1,2	1,2	1,3	1,4	1,5	62,2
Nordeste	1,4	1,4	1,5	1,5	1,6	1,7	1,8	1,8	1,9	1,9	38,5
Sudeste	2,6	2,7	2,7	2,8	2,9	2,9	2,9	3,0	3,1	3,2	22,4
Sul	2,3	2,2	2,2	2,3	2,3	2,3	2,4	2,4	2,5	2,6	14,1
Centro-oeste	2,1	2,2	2,3	2,4	2,5	2,7	2,7	2,8	2,9	3,1	45,6
Total	2,1	2,1	2,1	2,2	2,3	2,3	2,4	2,4	2,6	2,6	27,3
% de nefrologistas que atendem SUS											
Norte	91,0	88,6	90,2	89,3	87,0	88,1	83,2	83,6	84,7	87,6	-3,8
Nordeste	90,3	91,0	90,4	90,8	90,8	90,9	90,5	88,6	88,3	86,6	-4,1
Sudeste	82,9	81,1	79,6	79,1	78,0	77,9	76,8	74,0	73,6	72,7	-12,3
Sul	84,6	83,0	83,6	81,9	81,2	79,7	79,5	80,6	80,4	79,5	-6,0
Centro-oeste	82,4	82,5	85,9	86,3	82,2	83,7	85,0	84,0	83,0	82,5	0,1
Total	84,8	83,6	83,2	82,8	81,7	81,7	81,0	79,3	79,0	78,1	-7,9
% de nefrologistas em unidade de ensino											
Norte	58,3	55,4	55,7	51,8	48,8	49,3	47,8	43,9	47,8	48,9	-16,2
Nordeste	61,8	62,8	60,8	61,8	61,5	62,4	61,1	61,8	57,9	56,0	-9,4
Sudeste	51,3	49,7	49,5	49,3	47,5	47,2	46,6	43,2	42,7	42,1	-17,9
Sul	51,5	49,0	48,2	45,6	46,7	43,2	43,6	45,6	43,5	44,3	-14,0
Centro-oeste	44,4	42,9	42,9	45,0	52,7	43,9	45,4	42,9	45,2	44,4	0,2
Total	53,0	51,7	51,2	50,9	50,6	49,4	49,0	47,3	46,4	45,7	-13,7

Densidade de nefrologistas = número de nefrologistas registrados no CNES por 100.000 habitantes. SUS, Sistema Único de Saúde. CNES, cadastro nacional de estabelecimentos de saúde.

Referências

1. Brasil. Ministério da Saúde. DATASUS Tecnologia da Informação a Serviço do SUS. Cadastro Nacional de Estabelecimentos de Saúde. Recursos humanos. Profissionais segundo CBO [Internet]. 2025 [citado em 2025 maio 4]. Disponível em: http://tabnet.datasus.gov.br/cgi/deftohtm.exe?cnes/cnv/prid02br.def.
