# Supplementary material for: Coverage of procedures related to chronic kidney disease care in the Brazilian Unified Health System (SUS): analysis of the 2015–2024 decade
Source: J Bras Nefrol. 2026 Jan 23;48(2):e20250144. doi: 10.1590/2175-8239-JBN-2025-0144en (PMC12893125; doi:10.1590/2175-8239-JBN-2025-0144en)
Supplement: Translation

# Translation: Cobertura de procedimentos relacionados à assistência à doença renal crônica no Sistema Único de Saúde do Brasil: análise da década 2015–2024

**DOI:** 10.1590/2175-8239-JBN-2025-0144pt

**Authors:** Farid Samaan, Lan Hee Suh, Sônia Dias Lanza Freire, Ricardo Sesso, Gianna Mastroianni Kirsztajn

**Affiliations:** 1 Secretaria de Estado da Saúde de São Paulo, Coordenadoria de Regiões de Saúde, Grupo de Planejamento e Avaliação, São Paulo, SP, Brasil.; 2 Instituto Dante Pazzanese de Cardiologia, Divisão de Pesquisa, São Paulo, SP, Brasil.; 3 Universidade Federal de São Paulo, Disciplina de Nefrologia, São Paulo, SP, Brasil.

**Keywords:** Sistema Único de Saúde, Acesso a Serviços de Saúde, Sistemas de Informação em Saúde, Terapia de Substituição Renal, Doença Renal Crônica, Hemodiálise, Fístula Arteriovenosa

## Abstract

**Introdução:**

Globalmente, informações quantitativas sobre a cobertura assistencial à doença renal crônica (DRC) são escassas. Nosso objetivo foi estimar a razão oferta/necessidade de procedimentos relacionados à DRC no Sistema Único de Saúde do Brasil (SUS) entre 2015 e 2024.

**Métodos:**

O montante de exames, consultas e tratamentos relacionados à DRC foi consultado no endereço eletrônico do Departamento de Informática do SUS. Os parâmetros de necessidade desses procedimentos foram obtidos em portarias do Ministério da Saúde e revisão de literatura. A cobertura percentual de cada procedimento foi definida pela razão entre montante realizado e estimativa de necessidade.

**Resultados:**

A cobertura dos seguintes procedimentos aumentou entre 2015-2024: dosagem de creatinina sérica (70% a 122%), proteinúria (4% a 12%), ultrassonografia renal (76% a 107%), consulta ambulatorial com nefrologista (48% a 164%), acompanhamento multiprofissional da DRC pré-diálise (0% a 3%) e diálise crônica (69% a 81%). A cobertura de biópsia renal permaneceu praticamente estável (19% a 21%). Houve redução da cobertura de fístula arteriovenosa para hemodiálise (HD) (66% a 59%) e de transplante renal (46% a 37%). A utilização de diálise peritoneal (DP), entre os métodos de diálise crônica (DP e HD), caiu de 7% para 4%.

**Conclusão:**

Possíveis explicações para esses resultados são realização excessiva de dosagens de creatinina e de consultas com nefrologista, negligência do rastreamento da DRC por proteinúria, falta de adesão ao acompan­hamento multiprofissional pré-diálise, subutilização de DP e oferta insuficiente de biópsia renal e de terapia renal substitutiva (menor cobertura de transplante renal do que de diálise crônica).

## Introdução

A doença renal crônica (DRC) é um dos melhores modelos de prevenção em saúde, uma vez que seus principais fatores de risco são conhecidos (hipertensão arterial, diabetes mellitus, envelhecimento, obesidade), o diagnóstico precoce é simples e de baixo custo, e muitas ações de prevenção primária, secundária e terciária são factíveis e custo-efetivas^1,2^.

Paradoxalmente, a DRC é a doença crônica não transmissível mais negligenciada do mundo, sobretudo em países de rendas baixa e média^3^. Dentre essas nações, destaca-se o Brasil, país latino-americano em franca transição epidemiológica e demográfica e com a quarta maior população em programas de diálise crônica do mundo^4^. Nas últimas décadas, a prevalência dos principais fatores de risco para DRC apresentou elevação significativa no Brasil: obesidade, de 12% para 23%; hipertensão arterial (HA), de 20% para 28%; diabetes (DM), de 5 para 8%; e idade acima de 60 anos, de 9 para 13%^5,6^.

A agenda global de enfrentamento da DRC envolve, além do treinamento profissional e de campanhas de conscientização de pacientes, a ampliação da cobertura assistencial^1,3^. Para essa última ação, determinar as estimativas de necessidade de cada população e monitorar a oferta de serviços de saúde é fundamental.

No Brasil, a metodologia de cálculo de necessidade de serviços no Sistema Único de Saúde (SUS) utilizou como base evidências científicas nacionais e internacionais, pareceres de especialistas e consulta pública, culminando na Portaria de Parâmetros Assistenciais para Planejamento e Programação de Ações e Serviços de Saúde no SUS^7^. Globalmente, informações quantitativas sobre a cobertura da assistência à DRC são escassas. Assim, o objetivo deste estudo foi estimar a razão oferta/necessidade de procedimentos relacionados à DRC no Sistema Único de Saúde do Brasil (SUS) entre 2015 e 2024.

## Métodos

### Delineamento do Estudo

Trata-se de estudo descritivo realizado com base nos sistemas de informações ambulatoriais (SIA/SUS) e hospitalares (SIH/SUS) do SUS^8^. O SIA e o SIH são bases de dados secundários que armazenam informações de procedimentos relacionados à assistência à saúde (consultas, exames, medicamentos de alto custo, cirurgias e outras terapias, internações, entre outros). Esses sistemas têm abrangência nacional, englobam todos os estabelecimentos de saúde habilitados para prestar serviço ao SUS, e seu objetivo é de cobrança. As informações sobre o montante de procedimentos realizados são enviadas mensalmente, por via eletrônica, pelos estabelecimentos de saúde ao Ministério da Saúde, o qual consolida e publica os dados no site do DATASUS em 60–90 dias. Esses dados são de domínio público e permitem estratificações por município, micro e macrorregiões de saúde dos estados e regiões do Brasil.

O período analisado foi de 01/01/2015 a 31/12/2024, tendo como local o Brasil, estratificado por regiões geográficas. A extração dos dados dos sistemas de informação ocorreu entre os meses de janeiro e março de 2025. Este estudo está isento de apreciação por Comitê de Ética em Pesquisa por utilizar exclusivamente dados secundários, de acesso público, com informações agrupadas e sem possibilidade de acesso a dados individuais.

### Variáveis de Interesse

Os procedimentos relacionados à assistência à DRC consultados para este estudo foram: dosagem de creatinina sérica, dosagem de albumina na urina, dosagem de proteinúria de 24 horas, ultrassonografia renal, consulta médica com nefrologista, biópsia renal, acompanhamento multiprofissional da DRC, confecção de fístula arteriovenosa, hemodiálise, diálise peritoneal e transplante renal^8^. A escolha desses procedimentos foi realizada de acordo com os seguintes critérios: relevância científica, disponibilidade das informações em bancos de dados secundários do SUS e presença de estimativa de necessidade em diretrizes vigentes, portarias mini­steriais ou em revisões de literatura (Tabela 1)^7–12^. A lista detalhada desses procedimentos, com os respectivos códigos do Sistema de Gerenciamento da Tabela de Procedimentos, Medicamentos e OPM (Órteses, Próteses e Materiais) do SUS (SIGTAP)^13^, está apresentada na Tabela S1.

**Tabela 1:** Parâmetros de necessidade de procedimentos relacionados à assistência à doença renal crônica

Procedimento		Parâmetro de necessidade
Exames complementares	Dosagem de creatinina sérica	1 dosagem/ano por adulto com hipertensão arterial e/ou diabetes mellitus^9^
	Dosagem de proteinúria	Somatória das dosagens de albuminúria e proteinúria = 1 dosagem/ano por adulto com hipertensão e/ou diabetes^9^
	Ultrassonografia renal	800 exames/ano por 100.000 habitantes^7^
	Biópsia renal	10 biópsias/ano por 100.000 habitantes^10^
Consultas	Consulta com nefrologista	1600 consultas/ano por 100.000 habitantes^7^
	Acompanhamento multiprofissional da DRC	5 procedimentos/ano por adulto com DRC estágios 4 ou 5 não-dialítico^7,13^
Procedimentos cirúrgicos	Fístula arteriovenosa para hemodiálise	Número de FAV confeccionadas/mês = 5% do número de pacientes em hemodiálise^11^
Terapia renal substitutiva	Diálise crônica (hemodiálise e diálise peritoneal)	Prevalência de pacientes em diálise crônica com idade ≥20 anos, segundo regiões do Brasil: Norte: 0,08%; Nordeste: 0,11%; Sudeste: 0,13%; Sul: 0,11%; Centro-Oeste: 0,13%; Brasil: 0,12%^7^
	Transplante renal	Número de transplantes renais/ano = 10% do número de pacientes prevalentes em diálise crônica^12^

Abreviaturas – DRC: doença renal crônica; FAV: fístula arteriovenosa.

O percentual de cobertura de cada procedimento foi definido pela razão entre o montante realizado e a estimativa de necessidade. Os resultados de cobertura dos exames, consultas e tratamentos avaliados foram calculados anualmente e estratificados segundo a região geográfica do Brasil. A variação (*Δ*) entre o montante realizado e as coberturas de cada procedimento foi calculada em dois momentos (anos 2015 e 2024), de modo relativo (*Δ* relativa, em %) e por meio da fórmula:

*Δ* (%) = (resultado de 2024 – resultado de 2015)* 100/resultado de 2015

### População de Referência

A população de referência para as estimativas das coberturas de dosagem de creatinina sérica, proteinúria, ultrassonografia renal, consultas com nefrologista, biópsia renal e acompanhamento multiprofissional da DRC foi composta pelos indivíduos que dependem exclusivamente do SUS. Essa população foi calculada pela diferença entre a população total e o percentual de cobertura de saúde suplementar em cada região do Brasil^14^.

A população de referência para a estimativa das coberturas de diálise crônica e transplante renal foi o número de indivíduos que dependem exclusivamente do SUS, acrescido do percentual da população com cobertura de saúde suplementar que utilizou o SUS para esses procedimentos em cada região do Brasil. Esse fator de correção foi estimado a partir de informações de ressarcimento ao SUS fornecidas pela Agência Nacional de Saúde Suplementar (ANS)^14,15^ (Tabela S2). Para a diálise crônica, o uso do SUS por beneficiários de planos privados foi estimado em: 60% na Região Norte, 25% na Região Nordeste, 20% nas Regiões Sudeste e Sul, 15% na Região Centro-Oeste e 20% no Brasil como um todo; para os transplantes renais, o percentual estimado foi: 90% na Região Norte, 70% na Região Nordeste, 40% na Regiões Sudeste e Sul, 45% na Região Centro-Oeste e 45% no Brasil como um todo.

A população de adultos com hipertensão arterial e/ou diabetes mellitus, utilizada na análise de cobertura de dosagens de creatinina sérica e proteinúria, foi estimada em 30%, a partir de uma prevalência de HA de 29%, DM de 9% e da combinação de ambas, de 8%^5^. Os indivíduos com DRC estágios 4 e 5 não dialítica foram estimados em 0,2% da população de adultos^7^.

A população de referência para a estimativa da cobertura de fístula arteriovenosa foi o número de indivíduos em hemodiálise. O número médio de pessoas prevalentes em diálise (HD e DP) em cada ano foi estimado a partir do montante de procedimentos realizados no SUS^8^ (Tabela S3). Especificamente, no caso da diálise peritoneal (DP), estimou-se sua utilização no SUS (%) por meio da fórmula: número de pessoas em DP*100/número total de pessoas em diálise crônica (HD + DP).

### Análise Estatística

As variáveis de interesse, extraídas dos sistemas de informações do SUS, foram registradas em programa de planilhas (software Microsoft Excel, versão 2019). Nesse mesmo programa, foram calculados o número de adultos com HA e/ou DM, o número de procedimentos necessários (de acordo com os parâmetros pré-estabelecidos) e as respectivas coberturas (razões de oferta/necessidade).

## Resultados

Entre 2015 e 2024, a população total brasileira passou de 202.403.642 para 212.583.750, o que correspondeu a um aumento de 5,0%. O maior aumento populacional foi observado na Região Centro-Oeste (+10,8%) e o menor, na Região Nordeste (+3,5%). No mesmo período, o número de habitantes com idade igual ou superior a 20 anos apresentou elevação de 11,3% no Brasil, sendo a maior elevação na Região Norte (+18,6%) e a menor, na Região Sudeste (+8,8%). A população de indivíduos com idade ≥ 60 anos aumentou 37,1% no Brasil, sendo o maior aumento na Região Centro-Oeste (+48,6%) e o menor, na Região Nordeste (+32,7%). A cobertura de saúde suplementar no Brasil apresentou ligeira queda entre 2015 e 2024, passando de 24,7% para 24,0% (redução de 2,8%). As maiores reduções aconteceram nas regiões Norte (*−*10,5%) e Sudeste (*−*7,8%) (Tabela S4).

Entre 2015 e 2024, o montante de dosagens de creatinina sérica apresentou elevação de 31.341.496 para 55.671.370 (+77,6%); a maior elevação relativa ocorreu na Região Norte (+110,1%) e a menor, na Região Sudeste (+66,7%). No mesmo período, o número de dosagens de proteinúria (albumina urinária e proteinúria de 24 horas) aumentou de 1.246.144 para 4.069.988 (+226,6%); o maior aumento relativo foi na Região Sudeste (+316,3%) e o menor, na Região Norte (+66,4%). O montante de ultrassonografias renais apresentou elevação de 928.175 para 1.384.854 (+49,2%); a maior elevação se deu na Região Nordeste (+92,7%) e a menor, na Região Sul (+30,8%). Entre 2015 e 2024, as consultas médicas com nefrologista aumentaram de 1.159.174 para 4.246.100 (elevação de 266,3%); o maior aumento relativo foi na Região Nordeste (+535,0%) e o menor, na Região Norte (+72,2%). No mesmo período, o montante de biópsias renais apresentou elevação de 2.834 para 3.393 (+19,7%); o maior aumento ocorreu na Região Norte (+218,2%) e houve queda desse número na Região Centro-Oeste (*−*25,3%). As fístulas arteriovenosas confeccionadas foram 34.430 em 2015 e 40.334 em 2024 (+17,1%); a maior elevação relativa foi na Região Norte (+57,0%) e a menor, na Região Sul (+1,6%). O número de transplantes renais realizados em 2015 foi 4.925 e em 2024, 5.065 (+2,8%); o maior aumento relativo ocorreu na Região Centro-Oeste (+112,7%), e duas regiões apresentaram queda desse número: Nordeste (*−*5,9%) e Sudeste (*−*2,1%) (Tabela 2).

**Tabela 2:** Número de procedimentos relacionados à assistência à DRC financiados pelo SUS, segundo regiões do Brasil (2015–2024)

Procedimento (n) Região	2015	2016	2017	2018	2019	2020	2021	2022	2023	2024	Δ Rel. (%)
Creatinina sérica											
Norte	1.959.000	2.004.077	2.060.957	2.280.767	2.469.609	2.172.184	3.021.523	3.569.288	3.867.777	4.116.055	+110,1
Nordeste	5.980.782	6.043.061	6.588.021	7.287.783	7.809.865	6.453.628	8.553.296	9.622.308	10.542.531	11.501.585	+92,3
Sudeste	16.457.313	16.703.581	16.883.552	18.712.399	19.925.259	16.607.054	20.816.569	23.181.565	25.733.498	27.441.005	+66,7
Sul	4.915.068	5.077.338	5.421.119	5.815.820	6.268.103	5.093.574	6.164.617	7.125.974	7.978.346	8.486.227	+72,7
Centro-Oeste	2.029.333	2.180.719	2.422.533	2.454.803	2.852.839	2.310.253	3.040.985	3.566.131	3.837.401	4.126.498	+103,3
Total	31.341.496	32.008.776	33.376.182	36.551.572	39.325.675	32.636.693	41.596.990	47.065.266	51.959.553	55.671.370	+77,6
Proteinúria											
Norte	126.219	150.350	158.877	141.461	148.971	144.750	163.753	166.378	168.571	209.994	+66,4
Nordeste	177.701	179.294	193.342	219.746	278.000	235.802	313.358	356.767	424.323	496.200	+179,2
Sudeste	532.648	627.917	650.542	781.909	920.051	761.313	1.000.185	1.250.417	1.568.906	2.217.237	+316,3
Sul	289.488	285.489	306.658	362.521	422.037	367.553	471.259	606.577	779.328	916.493	+216,6
Centro-Oeste	120.088	105.055	136.788	149.037	154.748	127.414	181.539	189.128	218.344	230.064	+91,6
Total	1.246.144	1.348.105	1.446.207	1.654.674	1.923.807	1.636.832	2.130.094	2.569.267	3.159.472	4.069.988	+226,6
US renal											
Norte	54.741	51.882	52.878	56.697	59.085	38.525	48.170	63.237	74.035	83.973	+53,4
Nordeste	172.199	177.627	188.235	210.149	212.291	135.877	183.296	227.522	265.923	331.789	+92,7
Sudeste	503.938	513.675	544.401	563.510	591.578	422.830	515.732	575.068	640.178	699.522	+38,8
Sul	132.028	133.878	139.035	146.265	161.610	121.387	144.715	163.257	173.929	172.685	+30,8
Centro-Oeste	65.269	63.735	61.956	69.545	70.248	52.718	62.800	80.762	87.409	96.885	+48,4
Total	928.175	940.797	986.505	1.046.166	1.094.812	771.337	954.713	1.109.846	1.241.474	1.384.854	+49,2
Consulta com nefrologista											
Norte	119.096	116.578	128.782	137.552	113.724	103.042	110.830	152.914	190.074	205.089	+72,2
Nordeste	207.000	225.990	441.690	505.634	542.138	511.599	603.500	742.637	1.212.544	1.314.516	+535,0
Sudeste	558.169	552.009	569.690	607.858	646.899	482.461	546.933	703.194	1.334.670	1.667.207	+198,7
Sul	199.890	199.202	201.303	227.994	239.429	189.246	232.248	267.510	480.326	754.427	+277,4
Centro-Oeste	75.019	97.200	107.770	112.481	108.660	89.539	105.571	132.371	228.066	304.861	+306,4
Total	1.159.174	1.190.979	1.449.235	1.591.519	1.650.850	1.375.887	1.599.082	1.998.626	3.445.680	4.246.100	+266,3
Biópsia renal											
Norte	77	106	100	107	144	132	135	155	237	245	+218,2
Nordeste	564	710	925	909	1.146	718	882	757	917	581	+3,0
Sudeste	1.463	1.564	1.493	1.441	1.612	1.290	1.365	1.393	1.673	1.821	+24,5
Sul	548	676	747	607	554	602	527	515	598	610	+11,3
Centro-Oeste	182	254	219	258	187	168	182	140	177	136	25,3
Total	2.834	3.310	3.484	3.322	3.643	2.910	3.091	2.960	3.602	3.393	+19,7
Ac. multip. DRC											
Norte	0	25	241	165	169	33	0	46	195	200	+2,6
Nordeste	0	0	0	0	38	44	1.056	2.272	3.588	5.513	+53,7
Sudeste	0	0	83	918	2.164	2.383	5.429	9.521	16.566	20.314	+22,6
Sul	0	0	0	174	282	519	739	3.343	6.590	11.363	+72,4
Centro-Oeste	0	0	31	286	569	579	560	752	1.011	1.152	+13,9
Total	0	25	355	1.543	3.222	3.558	7.784	15.934	27.950	38.542	+37,9
FAV											
Norte	1.610	1.599	2.109	2.013	2.101	2.058	2.620	2.568	2.696	2.528	+57,0
Nordeste	9.463	9.698	10.600	10.949	11.838	10.730	11.529	11.213	12.245	12.723	+34,4
Sudeste	15.386	15.286	15.230	16.527	17.613	16.900	16.431	17.274	17.576	16.351	+6,3
Sul	5.326	5.384	5.399	5.597	5.965	5.746	5.211	5.578	5.510	5.411	+1,6
Centro-Oeste	2.645	2.560	2.676	2.900	2.933	2.895	2.605	2.904	2.944	3.321	+25,6
Total	34.430	34.527	36.014	37.986	40.450	38.329	38.396	39.537	40.971	40.334	+17,1
Transplante renal											
Norte	79	119	90	70	72	16	31	39	116	145	+83,5
Nordeste	862	778	893	1.008	1.030	664	746	800	915	811	-5,9
Sudeste	2.677	2.585	2.768	2.688	2.898	2.305	2.401	2.553	2.745	2.620	-2,1
Sul	1.189	1.275	1.371	1.413	1.353	1.017	882	1.109	1.246	1.238	+4,1
Centro-Oeste	118	175	190	223	268	240	196	165	280	251	+112,7
Total	4.925	4.932	5.312	5.402	5.621	4.242	4.256	4.666	5.302	5.065	+2,8

Abreviaturas – DRC, doença renal crônica. SUS, Sistema Único de Saúde. US, ultrassonografia. Ac. Multip. DRC, acompanhamento multiprofissional da DRC. FAV, fístula arteriovenosa.

Notas – Δ Rel. (%), delta relativo: variação relativa entre 2015 e 2024 para todas as variáveis, exceto ac. multip. DRC (Δ Rel. [%] entre 2023 e 2024).

Entre 2015 e 2024, a população em diálise crônica no SUS passou de 93.085 pessoas para 121.914 (+31,0%); a maior elevação relativa foi na Região Norte (+60,4%) e a menor, na Região Sudeste (+19,0%). O número de pessoas na modalidade de HD aumentou de 86.755 para 116.658 (+34,5%) no SUS; o maior aumento relativo foi na Região Norte (+70,8%) e o menor, na Região Sudeste (+22,0%). No mesmo período, o número de pacientes em DP no SUS caiu de 6.331 para 5.256 (*−*17,0%); o local com a maior queda relativa foi a Região Nordeste (*−*59,4%), e a Região Centro-Oeste apresentou o maior aumento do número de pessoas em DP (+102,1%) (Tabela 3).

**Tabela 3:** Estimativa de pacientes em diálise crônica financiada pelo SUS, segundo regiões do Brasil (2015–2024)

Procedimento (n) Região	2015	2016	2017	2018	2019	2020	2021	2022	2023	2024	Δ Rel. (%)
HD											
Norte	4.008	4.185	4.810	5.135	5.477	5.790	5.868	6.270	6.482	6.848	+70,8
Nordeste	22.595	23.443	24.308	25.732	27.321	28.511	28.991	30.551	32.711	35.254	+56,0
Sudeste	41.718	42.660	43.057	44.419	45.858	47.004	46.417	47.121	49.228	50.895	+22,0
Sul	11.338	11.518	11.825	12.343	12.941	13.233	13.268	13.750	14.394	14.787	+30,4
Centro-Oeste	7.095	7.220	7.470	7.789	8.309	8.433	8.179	8.266	8.699	8.874	+25,1
Total	86.755	89.026	91.470	95.419	99.904	102.972	102.724	105.958	111.514	116.658	+34,5
DP											
Norte	358	298	226	204	190	165	161	151	166	154	-56,8
Nordeste	1.097	952	770	641	579	534	506	459	477	446	-59,4
Sudeste	3.473	3.434	3.313	3.236	3.243	3.278	3.086	2.896	2.893	2.862	-17,6
Sul	1.087	1.041	1.079	1.082	1.125	1.150	1.077	1.049	1.107	1.153	+6,1
Centro-Oeste	317	355	414	470	480	478	500	504	571	640	+102,1
Total	6.331	6.081	5.801	5.633	5.616	5.604	5.330	5.059	5.214	5.256	-17,0
HD + DP											
Norte	4.366	4.483	5.035	5.340	5.666	5.956	6.029	6.421	6.649	7.003	+60,4
Nordeste	23.692	24.395	25.078	26.373	27.899	29.045	29.497	31.011	33.187	35.700	+50,7
Sudeste	45.191	46.095	46.370	47.655	49.101	50.282	49.503	50.017	52.121	53.757	+19,0
Sul	12.425	12.559	12.904	13.425	14.065	14.383	14.345	14.799	15.501	15.941	+28,3
Centro-Oeste	7.412	7.575	7.884	8.259	8.788	8.911	8.679	8.770	9.270	9.514	+28,4
Total	93.085	95.107	97.272	101.052	105.520	108.577	108.054	111.018	116.728	121.914	+31,0

Abreviaturas – SUS, Sistema Único de Saúde. HD, hemodiálise. DP, diálise peritoneal.

Notas – Estimativa de pacientes prevalentes em diálise crônica (HD e DP) financiada pelo SUS. Δ Rel. (%), delta relativo: variação relativa entre 2024 e 2015.

Entre 2014 e 2025, o percentual de cobertura de dosagem de creatinina sérica aumentou de 69,6% para 121,8% no Brasil (elevação de 75,0%); o aumento relativo foi maior na Região Sul (+74,8%) e menor na Região Nordeste (+46,7%). No mesmo período, a cobertura de dosagem de proteinúria apresentou elevação de 3,9% para 11,5% no país (aumento de 190,9%); essa elevação foi maior na Região Sudeste (+266,1%) e menor na Região Norte (+38,5%). O percentual de cobertura de ultrassonografia renal aumentou de 76,1% para 107,1% no Brasil (elevação de 40,8%); o aumento relativo foi maior na Região Nordeste (+86,9%) e menor na Região Sul (+20,9%). A cobertura de consultas médicas com nefrologista apresentou elevação de 47,5% para 164,3% entre 2015 e 2024 (aumento de 245,6%); essa elevação foi maior na Região Nordeste (+515,9%) e menor na Região Norte (+57,0%). O percentual de cobertura de biópsias renais aumentou de 18,6% para 21,0% no país (elevação de 13,0%); esse aumento foi maior na Região Norte (+190,1%) e a região Centro-Oeste apresentou queda desse indicador em 32,3%. Em 2024, a cobertura do acompanhamento multiprofissional da DRC alcançou 3,3% no Brasil, sendo maior na Região Sul (6,4%), seguida pelas coberturas nas Regiões Sudeste (4,6%), Nordeste (1,5%), Centro-Oeste (1,2%) e Norte (0,2%). O percentual de cobertura de FAV caiu de 66,1% para 58,7% no país (redução de 11,3%); essa queda foi maior na Região Sul (*−*21,2%), e apenas uma região apresentou valor positivo desse indicador (Centro-Oeste, com +0,5%). Entre 2015 e 2024, a cobertura de diálise crônica no Brasil apresentou elevação de 69,1% para 80,8% (aumento de 16,9%); essa elevação foi maior na Região Nordeste (+35,8%) e menor na Região Sudeste (+5,9%). No mesmo período, o percentual de cobertura de transplantes renais caiu de 45,6% para 36,9% (redução de 19,2%); essa queda foi maior na Região Nordeste (*−*36,2%), e duas regiões tiveram aumento desse indicador: Centro-Oeste (+67,1%) e Norte (+18,8%) ( Figura 1 e Tabela S5).

**Figura 1:** Cobertura de procedimentos relacionados à assistência à DRC no SUS, segundo regiões do Brasil (2015–2024).

Entre 2015 e 2024, o uso de DP no SUS caiu de 6,8% para 4,3% no Brasil. As seguintes regiões apresentaram a mesma tendência de queda: Norte (*−*73,2%), Nordeste (*−*3,0%), Sudeste (*−*30,7%) e Sul (*−*17,3%). A única região com aumento desse indicador foi a Região Centro-Oeste (+57,3%) (Tabela 4).

**Tabela 4:** Percentual de utilização de diálise peritoneal no SUS, segundo regiões do Brasil (2015–2024)

Região (%)	2015	2016	2017	2018	2019	2020	2021	2022	2023	2024	Δ Rel. (%)
Norte	8,2	6,6	4,5	3,8	3,4	2,8	2,7	2,4	2,5	2,2	-73,2
Nordeste	4,6	3,9	3,1	2,4	2,1	1,8	1,7	1,5	1,4	1,2	-73,0
Sudeste	7,7	7,4	7,1	6,8	6,6	6,5	6,2	5,8	5,6	5,3	-30,7
Sul	8,7	8,3	8,4	8,1	8,0	8,0	7,5	7,1	7,1	7,2	-17,3
Centro-Oeste	4,3	4,7	5,3	5,7	5,5	5,4	5,8	5,7	6,2	6,7	+57,3
Total (%)	6,8	6,4	6,0	5,6	5,3	5,2	4,9	4,6	4,5	4,3	-36,6

Abreviaturas – SUS: Sistema Único de Saúde; DP: diálise peritoneal; HD: hemodiálise.

Notas – Utilização de diálise peritoneal (%) = número de pacientes em DP × 100/número de pacientes em diálise crônica (HD + DP). Δ Rel. (%), delta relativo: variação relativa entre 2024 e 2015.

## Discussão

Este estudo mostrou que, entre 2015 e 2024, houve elevação expressiva da cobertura de dosagem de creatinina sérica, ultrassonografia renal, consultas com nefrologista e diálise crônica no SUS. Em 2024, ainda persistiram déficits importantes nas coberturas de dosagem de proteinúria, acompanhamento multiprofissional da DRC pré-diálise, realização de biópsia renal, fístula arteriovenosa e transplante renal. As regiões Norte, Centro-Oeste e Nordeste, comparadas às regiões Sudeste e Sul, apresentaram maior carência de quase todos os procedimentos avaliados.

### Avanços do SUS

O SUS é uma das políticas públicas mais abrangentes do mundo^16^. Muitas ações do SUS são reconhecidas pela Organização Mundial da Saúde como exemplos para outros países, como o Programa Saúde da Família, o Programa Nacional de Imunizações, o acesso à terapia contra o vírus da imunodeficiência humana, entre outras^17–19^. Na assistência à DRC, o Brasil é um dos poucos países com cobertura universal de diálise crônica e transplante renal^20^. Entretanto, estudos brasileiros têm demonstrado que ainda existem falhas importantes no cuidado integral às pessoas sob risco de ou com DRC^21–23^.

### Diferenças Regionais

As diferenças regionais de cobertura da assistência em DRC observadas neste estudo possivelmente sofrem influência de condições sociais, econômicas, demográficas e epidemiológicas existentes em cada uma das regiões do país^24^. Sabe-se que idade avançada, HA, DM e obesidade são fatores de risco para DRC^1,9,25^. O percentual de idosos nas Regiões Norte, Centro-Oeste e Nordeste é menor que no Sudeste e no Sul^6^, o que implicaria menor demanda de saúde dessa população e, portanto, menores resultados calculados de cobertura. Considerando somente as capitais dos estados brasileiros, as prevalências de obesidade e DM apresentam menor variação regional, enquanto a prevalência de HA é, em geral, maior no Sudeste e Sul^5^. Infelizmente, não foi possível ajustar nossos resultados de cobertura por idade e perfil de comorbidades. Entretanto, fatores como renda, educação, saneamento básico, habitação, transporte, entre outros, são determinantes de saúde e de acesso a serviços de saúde tão ou mais importantes que a idade e o perfil de comorbidades^26^.

### Exames Complementares

Este estudo demonstrou que a cobertura de exames de proteinúria no Brasil (somatória das dosagens de albuminúria e proteinúria de 24 horas) é de apenas 12%, resultado corroborado por pesquisas anteriores^21–23,27^. Globalmente, a principal barreira para a melhora desse indicador é a falta de conhecimento da importância desse exame por parte dos profissionais de saúde e da população^1,3,28^. No caso do Brasil, trata-se de exame de baixo custo, amplamente disponível, cuja recomendação para ser realizado em pessoas de risco consta em diretrizes ministeriais há mais de uma década^9,13,29^. A presente análise mostrou taxas de cobertura de creatinina sérica e ultrassonografia renal acima de 100% em algumas regiões, o que poderia ser explicado, em parte, por repetição de exames em um mesmo indivíduo. É possível que esse aparente desperdício de dosagens de creatinina sérica e de ultrassonografia renal se deva à falta de integração entre os sistemas de informação e à fragmentação do cuidado à saúde no SUS^30^. Outro motivo seria a realização de exames desnecessários em pacientes sem fatores de risco para DRC; de fato, o conhecimento dos profissionais de saúde e da população sobre as fases iniciais da DRC é inferior a 20% e 5%, respectivamente^1,3,28^.

A baixa cobertura de biópsia renal observada neste estudo possivelmente se justifica pela baixa remuneração e oferta insuficiente, pois se trata de procedimento que demanda recursos físicos e humanos frequentemente indisponíveis em muitas regiões do Brasil (técnicas de imunofluorescência e microscopia eletrônica, médico especializado em patologia renal, entre outros)^10,13^. Considerando que a biópsia renal é solicitada por médico nefrologista, a referência tardia a este profissional contribui para menor solicitação desse exame, uma vez que a biópsia renal não é indicada para casos avançados de DRC^29,31^.

### Consultas com Nefrologista

É provável que o aumento expressivo da cobertura de consulta médica com nefrologista a partir de 2022 se deva parcialmente à autorização de alguns gestores estaduais para que os serviços de TRS cobrem do SUS uma consulta médica em cada sessão de hemodiálise realizada^32^. Assim, esse indicador deixaria de refletir as consultas ambulatoriais realizadas em pacientes do SUS que não estão em diálise. Além disso, o aparente excesso de consultas poderia ser explicado pelo encaminhamento de pacientes que poderiam estar sendo acompanhados somente na atenção primária à saúde^1,23^. Embora a densidade de nefrologistas tenha aumentado em todas as regiões do Brasil (número de profissionais por 100.000 habitantes), os percentuais desses profissionais que atuam no SUS e em instituições de ensino têm diminuído^33^. Essa tendência sugere um aumento da atuação dos médicos nefrologistas em serviços de saúde que atendem pessoas com cobertura de saúde privada (planos de saúde e/ou pagamento direto pelo paciente) (Tabela S6).

### Acompanhamento Multiprofissional da DRC

Os procedimentos de acompanhamento multiprofissional da DRC pré-diálise foram criados pelo Ministério da Saúde em 2014 com o objetivo de estimular clínicas de diálise a assistir esse grupo de pacientes, considerando a presença de profissionais especializados nesses locais^29^. Todavia, o financiamento federal para essa ação tem sido insuficiente^10^, e apenas poucos estados, que optaram por incentivos locais, têm obtido aumento de cobertura desses procedimentos^34,35^.

### Terapia Renal Substitutiva

A população em diálise estimada neste estudo é inferior aos dados dos inquéritos brasileiros de diálise da Sociedade Brasileira de Nefrologia (SBN)^36^. A principal explicação é que nosso estudo considerou somente procedimentos realizados no SUS, enquanto os censos da SBN incluíram indivíduos em diálise no SUS e na saúde suplementar. Além disso, este estudo baseou-se em procedimentos de TRS realizados apenas em serviços habilitados pelo Ministério da Saúde, podendo haver outros financiados exclusivamente com recursos estaduais ou municipais. O parâmetro de necessidade de diálise crônica utilizado considerou os indivíduos prevalentes em diálise, não os distinguindo, portanto, daqueles que iniciaram TRS recentemente.

Este estudo mostrou que, no Brasil, a cobertura de diálise crônica no ano de 2024 foi mais que o dobro da cobertura de transplante renal. Na última década, a oferta de assistência em diálise apresentou tendência de elevação em todas as regiões do Brasil, comparada à queda ou estagnação da cobertura de transplantes renais. Destaca-se que as desigualdades regionais têm sido progressivamente corrigidas na diálise crônica, mas que as diferenças de cobertura de transplante renal entre as regiões do país permanecem elevadas^37^.

Na última década, o aumento observado na cobertura de diálise crônica no Brasil deu-se exclusivamente às custas da elevação da prevalência de pessoas em HD. Esse resultado coincidiu temporalmente com a abertura do capital estrangeiro para investimentos na saúde^38^. Diferentemente do transplante renal, os serviços de diálise no Brasil têm como natureza jurídica predominante a de empresas privadas com fins lucrativos^8,36,39^. O uso da DP caiu em todas as regiões, exceto no Centro-Oeste, graças a incentivos locais^40^. Possíveis explicações são o subfinanciamento público em nível federal, a maior lucratividade para provedores de HD em relação à DP, a ausência de centros produtores de equipamentos e insumos distribuídos nas diferentes regiões do país, os custos excessivos com logística, a falta de serviços e de profissionais capacitados, entre outras^39^.

### Limitações

As limitações deste estudo devem ser reconhecidas. Primeiramente, os resultados não podem ser generalizados para indivíduos com saúde suplementar, os quais hoje são cerca de um quarto da população brasileira. Em segundo lugar, a utilização do SUS por pessoas com saúde suplementar foi considerada apenas para os procedimentos de TRS (diálise e transplante renal), não sendo possível obter informações sobre o número de pessoas com saúde privada que usaram o SUS para procedimentos de baixa e média complexidade (exames e consultas). Terceiro, os sistemas de informação não nos permitiram estratificar a cobertura de saúde suplementar e o montante de procedimentos de acordo com faixa etária e perfil de comorbidades. Em quarto lugar, não foi considerada a variação dos parâmetros de necessidade ao longo do tempo. Por fim, a metodologia empregada não nos permitiu avaliar eventuais duplicidades de procedimentos em um mesmo indivíduo.

## Conclusões

Este estudo utilizou essencialmente instrumentos que fazem parte da rotina de trabalho dos gestores do SUS. Foi possível identificar e quantificar diversas falhas na assistência pública e integral à DRC. Ao mesmo tempo em que se reconhece a demanda por novos recursos tecnológicos e pelo aperfeiçoamento e integração das bases de dados do SUS, as informações hoje disponíveis podem guiar ações para a ampliação da rede de serviços e corrigir as discrepâncias observadas.

## Data Availability

Os dados que suportam esse artigo estão disponíveis mediante solicitação com justificativa razoável ao autor correspondente.

## Material Suplementar

Tabela S1 – Códigos SIGTAP dos procedimentos relacionados à assistência à doença renal crônica consultados neste estudo.

Tabela S2 – Número de sessões de hemodiálise e de transplantes renais realizados no SUS por beneficiários de saúde suplementar (2013–2022).

Tabela S3 – Fórmulas de cálculo das estimativas de pessoas em diálise crônica, a partir do montante de procedimentos realizados.

Tabela S4 – Dados demográficos e cobertura de saúde suplementar, segundo regiões do Brasil (2015–2024).

Tabela S5 – Cobertura de procedimentos relacionados à assistência à DRC no SUS, segundo regiões do Brasil (2015–2024).

Tabela S6 – Densidade de nefrologistas e percentuais de atendimento desses profissionais no SUS e em instituições de ensino, segundo regiões do Brasil (2015–2024).
